# Supplementary figures and images for: Association between newborn separation, maternal consent and health outcomes: findings from a longitudinal survey in Kenya
Source: BMJ Open. 2021 Sep 28;11(9):e045907. doi: 10.1136/bmjopen-2020-045907 (PMC8479975; doi:10.1136/bmjopen-2020-045907)

## Supplement 1. Flowchart of baseline and follow-up analytic sample sizes

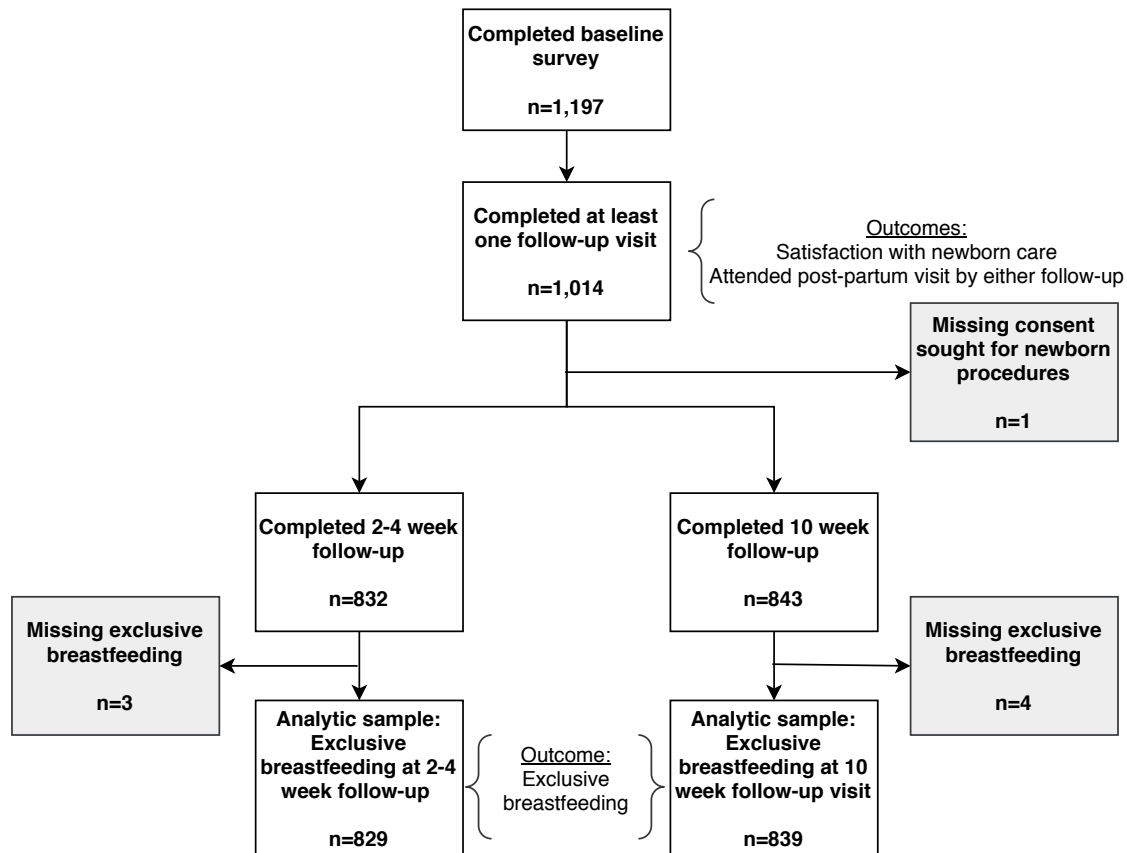

Supplement: Supplementary data [file bmjopen-2020-045907supp001.pdf]
