# Supplementary material for: Association between newborn separation, maternal consent and health outcomes: findings from a longitudinal survey in Kenya
Source: BMJ Open. 2021 Sep 28;11(9):e045907. doi: 10.1136/bmjopen-2020-045907 (PMC8479975; doi:10.1136/bmjopen-2020-045907)
Supplement: Supplementary data [file bmjopen-2020-045907supp002.pdf]

Supplement 2. Distributions of clinical and breastfeeding care indicators by reports of newborn separation and newborn consent indicators, (n=1,014)

Variable	Total	Newborn separation: Not separated	Newborn separation: Separated	Newborn separation: p-value	Newborn separation >10min: Not separated or separated ≤ 10min	Newborn separation >10min: Separated >10min	Newborn separation >10min: p-value	Consent sought for newborn procedures[1]: Did not ask for permission	Consent sought for newborn procedures[1]: Asked for permission	Consent sought for newborn procedures[1]: p-value
Total number in group	1,014	836	178		886	128		607	406	
**Clinical quality of care indicators**										
Was your baby put on your abdomen or chest as soon as it was born?										
No	13.6%	13.0%	16.3%	0.467	13.0%	18.0%	0.287	15.8%	10.3%	0.032
Yes	86.3%	86.8%	83.7%		86.9%	82.0%		84.0%	89.7%	
Don't know	0.1%	0.1%	0.0%		0.1%	0.0%		0.2%	0.0%	
Within two hours of delivery, did a health provider examine your baby?										
No	25.0%	25.7%	21.9%	0.049	26.4%	15.6%	0.001	31.3%	15.5%	<0.001
Yes	74.1%	73.7%	75.8%		73.0%	81.2%		67.5%	84.%	
Don't know	0.8%	0.6%	1.7%		0.6%	2.3%		1.2%	0.2%	
Missing	0.1%	0.0%	0.6%		0.0%	0.8%		0.0%	0.2%	
Was your baby wiped dry within a few minutes after birth?										
No	7.4%	7.3%	7.9%	0.013	7.2%	8.6%	0.091	9.2%	4.7%	0.003
Yes	90.4%	91.1%	87.1%		91.0%	86.7%		88.0%	94.3%	
Don't know	2.2%	1.6%	5.1%		1.8%	4.7%		2.8%	1.0%	
Has your baby been bathed yet?										
No	92.6%	93.3%	89.3%	<0.00	93.3%	87.5%	<0.00	92.6%	92.6%	0.723
Yes	5.8%	5.9%	5.6%		5.9%	5.5%		5.6%	6.2%	
Don't know	1.6%	0.8%	5.1%		0.8%	7.0%		1.8%	1.2%	
After birth, did any health care provider examine the cord?										
No	28.0%	27.4%	30.9%	0.344	27.9%	28.9%	0.809	34.8%	18.0%	<0.001
Yes	72.0%	72.6%	69.1%		72.1%	71.1%		65.2%	82.0%	
After birth, did any health care provider assess the temperature of your baby?										
No	51.1%	52.6%	43.8%	0.033	52.0%	44.5%	0.113	56.3%	43.1%	<0.001
Yes	48.9%	47.4%	56.2%		48.0%	55.5%		43.7%	56.9%	
After birth, did any health care provider counsel you on danger signs for newborns?										
No	63.4%	63.4%	63.5%	0.983	63.4%	63.3%	0.974	73.0%	49.0%	<0.001
Yes	36.6%	36.6%	36.5%		36.6%	36.7%		27.0%	51.0%	
**Breastfeeding care indicators**										
Within two hours of delivery, did a health provider check if breastfeeding was going well.										
No	24.2%	22.8%	30.3%	0.048	23.9%	25.8%	0.248	29.8%	15.8%	<0.001
Yes	75.6%	77.0%	69.1%		76.0%	73.4%		69.9%	84.2%	
Don't know	0.2%	0.1%	0.6%		0.1%	0.8%		0.3%	0.0%	
While you were in the hospital for the delivery of this baby did any one counsel/talk to you about breastfeeding?										
No	34.6%	33.5%	39.9%	0.103	34.%	39.1%	0.258	40.7%	25.4%	<0.001
Yes	65.4%	66.5%	60.1%		66.%	60.9%		59.3%	74.6%	
While you were in the hospital for the delivery of this baby did any one help you with breastfeeding by observing or showing you how to breastfeed?										
No	42.4%	40.6%	50.9%	0.013	41.5%	48.8%	0.126	51.1%	29.6%	<0.001
Yes	57.6%	59.4%	49.1%		58.5%	51.2%		48.9%	70.4%	

Note: For analyses, clinical quality and breastfeeding care indicators, “don’t know” responses were recoded as “no,” and one missing response was conservatively recoded as “yes.”

Nakphong MK, *et al. BMJ Open* 2022; 11:e045907. doi: 10.1136/bmjopen-2020-045907
