# Supplementary material for: Association between newborn separation, maternal consent and health outcomes: findings from a longitudinal survey in Kenya
Source: BMJ Open. 2021 Sep 28;11(9):e045907. doi: 10.1136/bmjopen-2020-045907 (PMC8479975; doi:10.1136/bmjopen-2020-045907)
Supplement: Supplementary data [file bmjopen-2020-045907supp003.pdf]

Results of selected multivariate logistic regression analyses (full models)

	Newborn separated for more than 10 minutes			Consent sought for newborn procedures								
	Exclusive breastfeeding at 2-4 week follow-up (n=829)			Satisfaction with newborn care at baseline (n=1,013)			Attend postpartum visit by either follow-up (n=1,013)			Exclusive breastfeeding at 10 week follow-up (n=839)		
Factor	**Coeff**	**95%CI**		**Coeff**	**95%CI**		**Coeff**	**95%CI**		**Coeff**	**95%CI**	
**Baby separated > 10 min (ref. not separated/separated <=10min)**	0.560***	(0.404,	0.776)	---			---			---		
**Consent sought for newborn procedures (ref. consent not sought)**	---			2.713***	(1.668,	4.411)	1.274*	(1.050,	1.546)	1.334**	(1.100,	1.619)
Age (continuous)	1.106***	(1.050,	1.165)	0.996	(0.948,	1.046)	1.048*	(1.010,	1.089)	1.046*	(1.002,	1.092)
Married (ref. not married)	0.634	(0.336,	1.199)	0.837	(0.568,	1.231)	0.942	(0.664,	1.336)	0.785	(0.408,	1.511)
Education (ref. No education/primary)												
Vocational/Secondary	1.093	(0.797,	1.498)	1.176	(0.714,	1.937)	1.084	(0.869,	1.353)	1.963**	(1.194,	3.226)
College/University	0.809	(0.513,	1.277)	1.324	(0.679,	2.584)	1.427	(0.927,	2.196)	2.306	(0.961,	5.532)
Employed (ref. not employed)	0.658**	(0.452,	0.957)	0.502*	(0.267,	0.945)	0.792*	(0.641,	0.979)	0.751	(0.561,	1.007)
Born in Nairobi or Kiambu counties (ref. woman born elsewhere)	1.117	(0.764,	1.632)	0.506	(0.274,	0.934)	1.352*	(1.071,	1.707)	0.910	(0.597,	1.387)
Multiparous (ref. primiparous)	1.420	(0.971,	2.076)	1.378	(0.839,	2.262)	1.087	(0.768,	1.539)	1.445	(0.803,	2.602)
Health status (ref. Excellent/Very good)												
Good	0.979	(0.701,	1.367)	1.311	(0.762,	2.257)	0.886	(0.731,	1.074)	0.864	(0.537,	1.390)
Fair	1.092	(0.453,	2.634)	1.667	(0.769,	3.614)	0.907	(0.657,	1.252)	0.857	(0.480,	1.532)
Poor or very poor	0.738	(0.340,	1.603)	8.688	(1.977,	38.186)	0.752	(0.524,	1.079)	1.443	(0.953,	2.186)
Facility level (ref. Gov't Hospital)												
Gov't HC/Disp	1.195	(0.618,	2.309)	1.770***	(1.528,	2.052)	1.139	(0.691,	1.879)	0.791	(0.556,	1.124)
Private Facility	0.547	(0.281,	1.063)	1.655	(0.878,	3.120)	1.707*	(1.087,	2.679)	0.492***	(0.334,	0.725)
Covered by health scheme or health insurance (ref. not covered)	1.401	(0.899,	2.184)	1.700	(0.823,	3.514)	0.989	(0.741,	1.321)	1.093	(0.793,	1.508)
Maternal Delivery Complications (ref. no complications)	---			0.673	(0.311,	1.454)	---			---		
Newborn Complications at baseline (ref. no complications)	0.441**	(0.264,	0.738)	1.094	(0.714,	1.678)	---			0.891	(0.399,	1.988)
Maternal Complications since discharge (ref. no complications)	0.557***	(0.434,	0.715)	---			1.650*	(1.060,	2.568)	0.662	(0.410,	1.069)
Newborn Complications since discharge (ref. no complications)	0.782	(0.396,	1.544)	---			---			0.898	(0.454,	1.776)
Newborn Complications at either baseline or since discharge (ref. no complications)	---			---			0.998	(0.608,	1.638)	---		
Clinical care Index (continuous)	1.112	(0.958,	1.290)	1.843***	(1.581,	2.149)	1.307***	(1.198,	1.425)	1.015	(0.838,	1.230)
Breastfeeding care Index (continuous)	1.164	(0.853,	1.590)	---			---			1.092	(0.897,	1.330)
Constant	0.621	(0.193,	1.994)	0.958	(0.355,	2.588)	0.0690***	(0.027,	0.175)	0.930	(0.382,	2.264)

*p<0.05, **p<0.01, ***p<0.001
