# Supplementary material for: Association between newborn separation, maternal consent and health outcomes: findings from a longitudinal survey in Kenya
Source: BMJ Open. 2021 Sep 28;11(9):e045907. doi: 10.1136/bmjopen-2020-045907 (PMC8479975; doi:10.1136/bmjopen-2020-045907)
Supplement: Author's manuscript [file bmjopen-2020-045907.draft_revisions.pdf]

BMJ Open is committed to open peer review. As part of this commitment we make the peer review history of every article we publish publicly available.

When an article is published we post the peer reviewers' comments and the authors' responses online. We also post the versions of the paper that were used during peer review. These are the versions that the peer review comments apply to.

The versions of the paper that follow are the versions that were submitted during the peer review process. They are not the versions of record or the final published versions. They should not be cited or distributed as the published version of this manuscript.

BMJ Open is an open access journal and the full, final, typeset and author-corrected version of record of the manuscript is available on our site with no access controls, subscription charges or pay-per-view fees (http://bmjopen.bmj.com).

If you have any questions on BMJ Open's open peer review process please email info.bmjopen@bmj.com

BMJ Open

Separating Newborns from Mothers and Maternal Consent for Newborn Care and the Association with Health Care Satisfaction, Use and Breastfeeding: Findings from a Longitudinal Survey in Kenya

Journal:	*BMJ Open*
Manuscript ID	bmjopen-2020-045907
Article Type:	Original research
Date Submitted by the Author:	15-Oct-2020

Complete List of Authors:	Nakphong, Michelle; University of California Los Angeles, Community Health Sciences<br>Sacks, Emma; Johns Hopkins University Bloomberg School of Public Health, Department of International Health<br>Opot, James; Innovations for Poverty Action<br>Sudhinaraset, May; University of California Los Angeles, Department of Community Health Sciences

Keywords:	OBSTETRICS, Health policy < HEALTH SERVICES ADMINISTRATION & MANAGEMENT, International health services < HEALTH SERVICES ADMINISTRATION & MANAGEMENT, Quality in health care < HEALTH SERVICES ADMINISTRATION & MANAGEMENT

SCHOLARONE™
Manuscripts

I, the Submitting Author has the right to grant and does grant on behalf of all authors of the Work (as defined in the below author licence), an exclusive licence and/or a non-exclusive licence for contributions from authors who are: i) UK Crown employees; ii) where BMJ has agreed a CC-BY licence shall apply, and/or iii) in accordance with the terms applicable for US Federal Government officers or employees acting as part of their official duties; on a worldwide, perpetual, irrevocable, royalty-free basis to BMJ Publishing Group Ltd ("BMJ") its licensees and where the relevant Journal is co-owned by BMJ to the co-owners of the Journal, to publish the Work in this journal and any other BMJ products and to exploit all rights, as set out in our licence.

The Submitting Author accepts and understands that any supply made under these terms is made by BMJ to the Submitting Author unless you are acting as an employee on behalf of your employer or a postgraduate student of an affiliated institution which is paying any applicable article publishing charge ("APC") for Open Access articles. Where the Submitting Author wishes to make the Work available on an Open Access basis (and intends to pay the relevant APC), the terms of reuse of such Open Access shall be governed by a Creative Commons licence – details of these licences and which Creative Commons licence will apply to this Work are set out in our licence referred to above.

Other than as permitted in any relevant BMJ Author's Self Archiving Policies, I confirm this Work has not been accepted for publication elsewhere, is not being considered for publication elsewhere and does not duplicate material already published. I confirm all authors consent to publication of this Work and authorise the granting of this licence.

For peer review only

Separating Newborns from Mothers and Maternal Consent for Newborn Care and the Association with Health Care Satisfaction, Use and Breastfeeding: Findings from a longitudinal survey in Kenya

Michelle K. Nakphong[1]*, Emma Sacks[2], James Opot[3], May Sudhinaraset[1]

[1] Department of Community Health Sciences, University of California, Los Angeles, CA, USA
[2] Department of International Health, Johns Hopkins School of Public Health, Baltimore, MD, USA
[3] Innovations for Poverty Action, Nairobi, Kenya

***Corresponding author:** Department of Community Health Sciences, University of California, Los Angeles; 650 Charles E. Young Drive South, 36-071 CHS, Box 951772 Los Angeles, CA 90095-1772. Email: hmkao@ucla.edu.

Word count: 3990

For peer review only

For peer review only - http://bmjopen.bmj.com/site/about/guidelines.xhtml

Separating Newborns from Mothers and Maternal Consent for Newborn Care and the Association with Health Care Satisfaction, Use and Breastfeeding: Findings from a Longitudinal Survey in Kenya

Abstract

Objectives Disrespectful and poor treatment of newborns such as unnecessary separation from parents or failure to obtain parental consent for medical procedures occurs at health facilities across contexts, but little research has investigated the prevalence, risk factors, or associated outcomes. This study aimed to examine these practices and associations with health care satisfaction, use, and breastfeeding.

Design Prospective cohort study

Setting Health facilities in Nairobi and Kiambu counties in Kenya

Participants Data were collected from women who delivered in health facilities between September 2019 and January 2020. The sample included 1,014 women surveyed at baseline and at least one follow-up at 2-4 or 10 weeks postpartum.

Primary and secondary outcome measures 1) Outcomes related to satisfaction with care and care utilization, 2) Continuation of post-discharge newborn care practices such as breastfeeding.

Results 17.6% of women reported being separated from their newborns at the facility after delivery, of whom 71.9% were separated over 10 minutes. 44.9% felt separation was unnecessary and 8.4% reported not knowing the reason for separation. 59.9% reported consent was not obtained for procedures on their newborn. Women separated from their newborn (>10 minutes) were 44% less likely to be exclusively breastfeeding at 2-4 weeks (aOR=0.56, 95%CI: 0.40, 0.76). Obtaining consent for newborn procedures corresponded with 2.7 times greater likelihood of satisfaction with care (aOR=2.71, 95%CI: 1.67, 4.41), 27% greater likelihood of postpartum visit attendance for self or newborn (aOR=1.27, 95%CI: 1.05, 4.41), and 33% greater likelihood of exclusive breastfeeding at 10 weeks (aOR=1.33, 95%CI: 1.10, 1.62).

Conclusions Newborns, mothers, and families have a right to high quality, respectful care, including the ability to stay together, be informed and have proper consent for care. The implications of these practices on health outcomes a month or more after discharge illustrate the importance of a positive experience of postnatal care.

Keywords

Newborn care; neonatal health; maternal child health; postnatal care; maternal-newborn separation; breastfeeding; respectful care; informed consent; quality of care; survey; observational data

For peer review only

Article Summary

Strengths and Limitations of the Study

- This is one of the first studies to include survey questions surrounding details of separation of newborns from mothers and maternal consent for care, including risk factors and reasons for separation.
- This study used longitudinal data collected over 10 weeks following delivery to assess associations with outcomes, including postpartum visit attendance and breastfeeding.
- Differences in participants who responded to the 2-4week and 10 week follow-up interviews may have introduced bias due to differences in composition, thus comparisons of associations with outcomes between these two points should be interpreted conservatively.
- Findings may have limited generalizability to other contexts, as the study sample included women who gave birth at facilities in Nairobi and Kiambu counties.

INTRODUCTION

The first month of life constitutes the most vulnerable time for a child's survival. Globally, 2.5 million children die within the first 28 days of life at a rate of 18 deaths per 1,000 live births.[1] Kenya has made notable progress in reducing neonatal mortality in recent years, [2] but with its current neonatal mortality ratio of 19.6 deaths per 1,000 live births, Kenya is unlikely to reach the 2030 Sustainable Development Goal target of 12 deaths per 1,000 live births.[3] Yet, substantial progress can still be made: over 80% of newborn deaths are considered preventable, highlighting the critical need to improve the quality of maternal and newborn care.[4]

Efforts to enhance the quality of maternity care have drawn attention to women's experience of care, including respect and dignity. The WHO Vision on Quality of Care for maternal and newborn health outlines two essential, interlinked dimensions of Quality of Care: provision of care and experience of care.[5] Integrally, quality care, in addition to being safe, effective,

For peer review only - http://bmjopen.bmj.com/site/about/guidelines.xhtml

timely, and efficient, must also be equitable and people-centered. Experience of care includes effective communication, respectful and dignified treatment, and access to emotional support. Evidence indicates that across contexts, many women experience mistreatment and abuse,[6–11] and lack access to the support person(s) of their choice during labor and delivery.[12–14] Negative experiences of care not only contribute to dissatisfaction with care,[15–17] but may deter women from seeking future health services.[18–22] However, the corresponding elements of quality newborn care and the health impact of these experiences have been under-researched.

WHO recommendations for newborn care include skin-to-skin care, early and exclusive breastfeeding, thermal and umbilical care, delayed bathing, and maintaining the newborn with family as much as possible.[23] These practices are especially important for preterm and low birth weight infants, who are more vulnerable to mortality and morbidity. It is also recommended that families receive breastfeeding support, counseling on danger signs, and exams of the infant and cord before discharge. Communication, consent, and respect for autonomy are important components of a positive experience of care. But, efforts to improve the quality of newborn care have often focused on provision of care with low or no priority on experience of care.[24] Research on experiences of newborn care (consent for procedures or exams, mistreatment and neglect, and separating infants from mothers or caregivers when not medically necessary) represents a notable gap in literature.

Evidence indicates that despite newborns' inability to exercise autonomy or verbally express needs, positive experiences of care center around their relationship and connection with their mothers and caregivers. A strong mother-infant relationship forms the foundation for infant

For peer review only

health and development, and the period after birth is especially important.[25] Immediately following birth, infants display highly specialized attachment and bonding behaviour towards their mothers,[26,27] and sensory connections with mothers form a strong basis for the regulation of their physiological systems.[28] For example, research on skin-to-skin contact (SSC) after birth has shown that early sensory connections improve many physical, psychological, and care outcomes, such as maintenance of newborn temperature,[30] stable respiratory and cardiac function,[31] organization of sleep cycles,[32] development of neurophysiological systems,[33,34] and reducing newborn stress.[30,35] In contrast, separation of mothers and infants hinders attachment, induces stress and behaviors that alter physiological processes resulting in an altered developmental trajectory.[28,29]

This highlights newborns' needs to effectively bond with family, receive attentive care, and be kept warm and treated gently. Sensory cues from infants–suckling, sight, smell, sounds–also stimulate maternal neurobiological processes which subsequently affect maternal mental health and caregiving. [37–39] Evidence also suggests that the benefits of early bonding likely extends to other caregivers as well, such as fathers, with implications for future involvement in care.[40]

Fostering the mother-infant bond after birth also has profound implications for early and long-term breastfeeding. Mother-infant interactions stimulate maternal milk production and prepare infants to breastfeed by downregulating the neuroendocrine stress response and activating blood glucose regulation.[41,42] The evidence of a link between close maternal-infant contact and breastfeeding is compelling: mother-infant pairs who engaged in SSC after birth were more likely to successfully breastfeed during their first feed, continue breastfeeding after 1-4 months

For peer review only

post-birth, score higher on breastfeeding effectiveness, and breastfeed exclusively and for longer durations when compared to those without SSC.[30,43] This underscores the need to foster mother-infant interactions and minimize unnecessary interventions in maternity care.

Lack of information and informed consent have been cited as factors contributing to traumatic experiences and dissatisfaction in maternity care.[44–46] Lack of consent for women's care is regarded as an essential component of respectful care, but consent for procedures performed on newborns has rarely been studied. Because infants are completely dependent upon their caregivers, caregivers are essential advocates to ensure safe and gentle medical care. Parents (or designated legal guardians) should be the decision-makers regarding their infants' care, and it is likely that women and families value the ability to make informed decisions and advocate for their newborns' care.

There are indications that poor treatment of newborns in facility-based maternity care occurs, including preliminary documentation of prevalence of certain types of occurrences. Recent literature has brought attention to the prevalence of practices such as unnecessary separation of newborns from mothers, neglect (absence of care), and non-consent for procedures.[7,10,47] However, these practices have seldom been examined as primary indicators and available information has emerged from secondary data from other studies. A multi-country African study on treatment of women included observations of newborns in the first two hours after birth and found that over half of newborns were separated from their mothers within the first two hours, with higher prevalence among single and less educated women.[48] However, while some studies have documented high prevalence of these practices and inequities in care, it remains

For peer review only - http://bmjopen.bmj.com/site/about/guidelines.xhtml

unclear how they are associated with other measures of quality care or outcomes (e.g. positive experiences of care, health behaviors, or health outcomes). Moreover, evidence suggests that person-centered maternity care is consequential for newborn health. One recent study in Kenya found that women's poor experiences of care were associated with increased report of newborn complications and lower likelihood of attending future medical visits.[52] To our knowledge, no previous studies have linked disrespectful neonatal care practices, namely separation of newborns from mothers and lack of parental consent for newborn procedures, to health outcomes. This study aims to address these gaps.

Further research is needed to explore how newborn care practices, and the implementation of institutional policies and provider practices, impact maternal and neonatal outcomes.[41,53] The aim of this study was to investigate the prevalence, risk factors, and outcomes associated with newborn care practices, namely separating newborns from mothers and parental consent for newborn care, in several facilities in Kenya. In this study, we examined these practices using data from the Strengthening Person-Centered, Accessibility, Respectful Care, and Quality Study (SPARQ) in Kenya. In addition to investigating the extent to which these practices occur, we sought to understand underlying risk factors and whether certain groups were at greater risk (i.e. 'discriminatory' practices). We also examined details surrounding newborn separation, including the duration and reasons for separation, and other newborn care practices such as SSC and breastfeeding support. Finally, we sought to understand the consequences of these practices on maternal and newborn health outcomes: satisfaction with care, postpartum care attendance, and breastfeeding.

For peer review only - http://bmjopen.bmj.com/site/about/guidelines.xhtml

METHODS

Setting

This longitudinal study was conducted between September 2019 and January 2020 across six health facilities within Nairobi and Kiambu Counties, Kenya. The facilities varied in type (four public and two private) and size (medium to large referral hospitals ranging from 100 to 900 reported deliveries per month). The facilities were conveniently selected based on their location within the two counties, reported deliveries of at least 100 per month, and the facility administration's willingness to participate in the study.

Data collection and recruitment

A team of twenty-four enumerators were involved in data collection, who had previous quantitative and research ethics training. The baseline survey was pre-tested with women who had recently delivered in four facilities (two of which were study facilities) to identify any issues with question flow or understanding via interviewing. The follow-up phone surveys were also pre-tested with this sample.

All surveys were administered in a private space within the facility to ensure confidentiality and privacy. Follow-up surveys were conducted by phone, ensuring privacy to maintain confidentiality.

Women were enrolled in the study postpartum when recovering in the maternity ward or at discharge before exiting the facility, all within 48 hours after birth. Women provided written

For peer review only

For peer review only - http://bmjopen.bmj.com/site/about/guidelines.xhtml

informed consent. Participants were also administered follow-up phone surveys between 2-4 weeks and/or 10 weeks postpartum. Participants were given airtime credit of approximately $2.00 for the baseline and $0.50 for each follow-up as a token of appreciation.

Analysis

The analytic sample included 1,014 women who completed baseline and at least one follow-up interview (Supplement 1). Data for exclusive breastfeeding were missing from 3 women in the 2-4 week follow-up and 4 women in the 10 week follow-up resulting in an analytic sample for exclusive breastfeeding of 829 and 839 participants in the 2-4 week and 10 week follow-up, respectively.

Variables examined are presented in Table 1. Our outcomes of interest included *Satisfaction with newborn care* measured at baseline, and *Attendance of post-partum check-up,* and *Exclusive breastfeeding* measured at follow-up. Primary independent variables were *Newborn separation, Newborn separation >10 minutes,* and *Consent for newborn procedures*. We examined descriptive characteristics of all women in the sample. We explored sociodemographic characteristics of the participants including age, parity, marital status, education, employment, woman's birthplace, insurance status, and facility type. We examined maternal health indicators of self-rated health status, maternal complications at delivery, and report of maternal complications since discharge. Newborn health characteristics were also explored, including birthweight, gestational age, and report of newborn complications at baseline and after discharge. Lastly, we examined other measures of care received using a *Clinical quality index* and a *Breastfeeding care index* based on WHO standards of care.[23]

For peer review only - http://bmjopen.bmj.com/site/about/guidelines.xhtml

Table 1. Definition of variables

Indicator	Question/Measure	Values
Outcome Indicators		
Satisfaction with newborn care	In general, how satisfied were you with the services and care your newborn received after delivery?	"Satisfied or very satisfied" vs. "Dissatisfied or very dissatisfied"
Attendance of post-partum check-up	Have you attended any postpartum visits since being discharged? [1]	"Yes" vs. "No"
Exclusive breastfeeding	How were you feeding your baby? [1]	Fed infant with breastmilk only vs. fed with any other substances
Newborn separation and consent indicators		
Newborn separation	Was your baby ever separated from you by doctors, nurses, or other health providers for any reason?	"Yes" vs. "No"
Newborn separation >10 minutes	Was your baby ever separated from you for more than 10 minutes?	"Yes" vs. "No"
Consent sought for newborn procedures	Did the doctors, nurses, or other providers ask you for your permission before doing procedures or examinations on your baby?	"Yes" vs. "No"
Other variables		
Clinical quality index	Summative score comprising 7 newborn care questions indicating whether specific procedures were performed (skin-to-skin contact after birth; infant exam after delivery; infant dried after birth; delayed bathing; cord examination; temperature assessment; and mother/family counseled on newborn danger signs)	Range 0-7
Breastfeeding care index	Summative score of 3 indicators of breastfeeding support at the facility. (i.e. provider checked breastfeeding within two hours of delivery; mother/family counseled about breastfeeding; brestfeeding observed or shown.	Range 0-3

[1]Postpartum visit could have been for either maternal or newborn health.

Bivariate analyses investigated relationships between newborn care indicators and outcomes using chi-square and t-tests. We conducted multivariate logistic regression analyses assessing associations between newborn separation and consent indicators and described outcomes. Analyses adjusted for factors that may theoretically confound associations including age, parity, marital status, education, employment, birthplace, health facility type, insurance status, self-rated health, and clinical care index score. Analyses for *Satisfaction with newborn care* also adjusted

For peer review only

for maternal and newborn complications at baseline and analyses for outcomes measured at follow-up interview were adjusted for maternal and newborn complications since discharge. Models for breastfeeding outcomes also adjusted for breastfeeding care index score. Cluster robust standard errors were used to account for clustering by facility. Model specification was tested using link tests and model fit was tested by Hosmer-Lemeshow goodness-of-fit tests. Sensitivity analyses examined potential effects of preterm birth and low birthweight, but no evidence of confounding was found. Analyses were performed using STATA/SE 15.1.

Ethics Approval

All study procedures were approved by the Institutional Review Boards at the University of California, San Francisco (protocol number 19-27783) and the Kenya Medical Research Institute (Protocol KEMRI Non-SSC 666). Informed consent was obtained from all participants prior to participation.

Patient and public involvement

Patients and the general community were not involved in the design, recruitment, or conduct of this study. During the consent process, participants were informed that they would not directly benefit from their participation or be involved in the dissemination of the study results. Study results will be shared with facilities in order to improve their service delivery.

RESULTS

For peer review only

Descriptive characteristics of the sample of 1,014 women, stratified by reports of *newborn separation, newborn separation >10 minutes, and consent sought for newborn procedures,* are presented in Table 2.

Women who were separated from their newborn more than 10 minutes tended to be slightly older (p=0.036) and not born in Nairobi or Kiambu counties (p=0.043) compared to women reporting separation for less than 10 minutes or no separation. A greater proportion of women at government hospitals were separated from their newborns compared to women at government health clinics or private facilities (p<0.001). Delivery complications were also positively associated with newborn separation (p=0.004) and separation for more than 10 minutes (p=0.025).

Women who were asked permission for newborn procedures and examinations were older (p=0.026) and more likely to be covered under a health scheme or health insurance (p=0.028) than those who were not asked for permission.

Table 2. Descriptive characteristics of women included in the sample by reports of newborn separation, newborn separation for more than 10 minutes, and consent sought for newborn procedures (n=1,014)

		Newborn separated			Newborn separated >10min			Consent for newborn procedures[1]		
**Characteristics**	**Total**	**Not separated**	**Separated**	**p-value**	**Not separated/ separated ≤10min**	**Separated >10min**	**p-value**	**Did not ask for permission**	**Asked for permission**	**p-value**
Total number in group	1014	836	178		886	128		607	406	
Age (mean)	25.7	25.58	26.11	0.202	25.55	26.54	0.036	25.38	26.1	0.026
SD	(5.01)	(4.97)	(5.19)		(4.95)	(5.32)		(4.96)	(5.05)	
Parity (mean)	2.0	2.00	2.16	0.050	2.01	2.22	0.021	2.02	2.04	0.714
SD	(0.98)	(0.97)	(1.02)		(0.97)	(1.03)		(0.98)	(0.98)	
Multiparous										
No	35.7%	36.7%	30.9%	0.141	36.7%	28.9%	0.086	36.1%	35.2%	0.780

For peer review only

For peer review only - http://bmjopen.bmj.com/site/about/guidelines.xhtml

		Newborn separated			Newborn separated >10min			Consent for newborn procedures[1]		
**Characteristics**	**Total**	**Not separated**	**Separated**	**p-value**	**Not separated/ separated ≤10min**	**Separated >10min**	**p-value**	**Did not ask for permission**	**Asked for permission**	**p-value**
Yes	64.3%	63.3%	69.1%		63.3%	71.1%		63.9%	64.8%	
Currently married or partnered										
No	81.8%	18.7%	16.3%	0.458	18.2%	18.8%	0.874	19.1%	17.0%	0.393
Yes	18.2%	81.3%	83.7%		81.8%	81.2%		80.9%	83.0%	
Educational attainment										
Primary or less	43.7%	43.8%	43.3%	0.894	44.0%	41.4%	0.855	44.8%	41.9%	0.267
Vocational/	39.6%	39.4%	41.0%		39.4%	41.4%		40.0%	39.2%	
College/University	16.7%	16.9%	15.7%		16.6%	17.2%		15.2%	19.0%	
Currently employed										
No	59.5%	59.0%	61.8%	0.486	59.7%	57.8%	0.683	61.6%	56.4%	0.098
Yes	40.5%	41.0%	38.2%		40.3%	42.2%		38.4%	43.6%	
Born in Nairobi or Kiambu Counties										
No	78.0%	76.8%	83.7%	0.043	77.3%	82.8%	0.160	77.6%	78.6%	0.713
Yes	22.0%	23.2%	16.3%		22.7%	17.2%		22.4%	21.4%	
Self-rated health status										
Excellent or very good	34.7%	33.5%	40.4%	0.043	34.1%	39.1%	0.144	33.3%	36.9%	0.502
Good	40.4%	42.5%	30.9%		41.6%	32.0%		40.4%	40.4%	
Fair	15.7%	15.2%	18.0%		15.0%	20.3%		16.8%	14.0%	
Poor or very poor	9.2%	8.9%	10.7%		9.3%	8.6%		9.6%	8.6%	
Maternal Complications during delivery										
No	93.6%	94.6%	88.8%	0.004	94.2%	89.1%	0.025	92.9%	94.6%	0.289
Yes	6.4%	5.4%	11.2%		5.8%	10.9%		7.1%	5.4%	
Reported maternal complications at follow-up										
No	86.1%	86.4%	84.8%	0.592	86.0%	86.7%	0.827	85.3%	87.2%	0.403
Yes	13.9%	13.6%	15.2%		14.0%	13.3%		14.7%	12.8%	
Covered under health scheme or health insurance										
No	14.0%	13.9%	14.6%	0.799	13.9%	14.8%	0.77	16.0%	11.1%	0.028
Yes	86.0%	86.1%	85.4%		86.1%	85.2%		84.0%	88.9%	
Facility type										
Gov't hospital	73.9%	70.8%	88.2%	<0.001	71.7%	89.1%	<0.001	74.3%	73.2%	0.881
Gov't Health Centre/Dispensary	12.0%	13.9%	3.4%		13.4%	2.3%		12.0%	12.1%	
Private facility	14.1%	15.3%	8.4%		14.9%	8.6%		13.7%	14.8%	

[1] N=1,013 (1 refused to answer)

Table 3 displays newborn characteristics and other clinical and breastfeeding care indicators by reported experience of newborn separation and consent sought for newborn care. 17.6% of women reported being separated from their newborns while at the health facility. Among those women (n=178), the majority reported separation longer than 10 minutes (71.9%) and nearly half felt separation was unnecessary (44.9%). Most of those separated reported that their newborn

For peer review only

was separated for procedures or examination (82.6%), but 8.4% reported that they did not know or were not told.[1] During separation, only one-in-five (19.6%) were able to have another parent, family member, or caregiver remain present with the newborn. Almost two-thirds of women (59.9%) reported that health providers did not ask permission before performing procedures or examinations on their newborn.

More newborn complications were reported among those separated than those who were not ($p<0.001$). Mothers who were separated from their newborns were more likely to have their infant examined after delivery than not ($p=0.049$) but were less likely to have a provider: ask consent before newborn procedures or exams ($p=0.023$), check if breastfeeding was going well ($p=0.048$), or observe or demonstrate how to breastfeed ($p=0.013$). Almost all indicators of better clinical and breastfeeding care, such as skin-to-skin contact, newborn exam after delivery, newborn wiped, cord examination, temperature assessment, counseling on danger signs for newborns, and breastfeeding checks, counseling, and observation, were positively associated with consent sought for newborn procedures (Supplement 2). Among the full sample, the mean clinical quality index score was 4.14 (SD 1.37) out of 7. Those who were asked permission for newborn procedures had a mean clinical quality of care index of 4.64 (SD 1.17) compared to 3.81(SD 1.38) among those who were not asked permission ($p<0.001$). The mean breastfeeding care index score among those who were asked permission was 2.3 (SD 0.94) out of 3 compared to 1.79 (SD 1.10) for those who were not asked permission ($p<0.001$).

Table 3. Distributions of newborn characteristics and newborn care indicators by reports of newborn separation and newborn consent indicators (n=1,014)

[1] Of those who reported "Don't know," 80.0% reported being satisfied with newborn care. In comparison, 94.1% of the total sample reported being satisfied with newborn care.

For peer review only - http://bmjopen.bmj.com/site/about/guidelines.xhtml

Variable	Total	Newborn separation: Not separated	Newborn separation: Separated	Newborn separation: p-value	Newborn separation >10min: Not separated or separated ≤10min	Newborn separation >10min: Separated >10min	Newborn separation >10min: p-value	Consent sought for newborn procedures[1]: Did not ask for permission	Consent sought for newborn procedures[1]: Asked for permission	Consent sought for newborn procedures[1]: p-value
Total number in group	1014	836	178		886	128		607	406	
**Health indicators**										
Birth weight										
Low BW (<2.5kg)	2.3%	2.2%	2.8%	0.002	2.3%	2.3%	<0.001	2.8%	1.5%	0.588
Normal BW (2.5-4.0kg)	89.3%	90.8%	82.6%		90.6%	80.5%		88.8%	90.2%	
Macrosomia (>4.0kg)	2.2%	2.2%	2.3%		2.1%	2.3%		1.7%	3.0%	
Missing	6.2%	4.9%	12.4%		5.0%	14.8%		6.8%	5.4%	
Preterm										
Not preterm (GA 38 weeks or more)	77.4%	77.2%	78.7%	0.418	77.3%	78.1%	0.266	77.9%	76.9%	<0.001
Yes, preterm (GA 37 weeks or less)	22.4%	22.7%	20.8%		22.6%	21.1%		21.9%	23.2%	
Missing	0.2%	0.1%	0.6%		0.1%	0.8%		0.2%	0.0%	
Newborn complications reported at baseline										
Healthy, no issues	96.3%	97.5%	90.5%	<0.001	97.5%	87.5%	<0.001	96.4%	96.1%	0.929
Complications	3.6%	2.4%	9.6%		2.4%	12.5%		3.5%	3.9%	
Missing[2]	0.1%	0.1%	0.0%		0.1%	0.0%		0.2%	0.0%	
Newborn complications reported since discharge										
No complications	67.8%	68.2%	65.7%	0.525	68.1%	65.6%	0.582	68.0%	67.2%	0.790
Complications	32.3%	31.8%	34.3%		31.9%	34.4%		32.0%	32.8%	
**Newborn care indicators**										
Newborn separation										
No	82.4%	---	---		---	---		80.9%	85.%	0.093
Yes	17.6%	---	---		---	---		19.1%	15.%	
Consent sought for newborn procedures										
No	59.9%	58.7%	65.2%	0.023	58.9%	66.4%	0.007	---	---	
Yes	40.0%	41.3%	34.3%		41.1%	32.8%		---	---	
Refused to answer	0.1%	0.0%	0.6%		0.0%	0.8%		---	---	
**Of those who were separated from newborns (n=178)**										
Newborn separation > 10 min										
No	4.9%	---	28.1%		---	---		26.7%	31.1%	0.534
Yes	12.6%	---	71.9%		---	---		73.3%	68.9%	
Was your baby separated from you by doctors, nurses, or other health providers to perform procedures and examinations?										
No	1.6%	---	9.0%		14.0%	7.0%	0.344	8.6%	9.8%	0.868
Yes	14.5%		82.6%		78.0%	84.4%		82.8%	83.6%	
Don't know	1.5%		8.4%		8.0%	8.6%		8.6%	6.6%	
Did you ever feel that it was not necessary for your baby to be separated from you?										
No	9.7%	---	55.1%		60.0%	53.1%	0.407	53.4%	59.0%	0.479
Yes	7.9%	---	44.9%		40.0%	46.9%		46.6%	41.0%	
If you were ever unable to be with your newborn, was another parent, family members, or caregiver able to be with your baby?										
No	14.2%	---	80.9%		78.0%	82.%	0.539	87.1%	68.9%	0.003
Yes	12.6%	---	19.1%		22.0%	18.%		12.9%	31.1%	
**Other care indicators**										
Clinical quality index										
Mean	4.14	4.14	4.15	0.965	4.13	4.20	0.637	3.81	4.64	<0.001

For peer review only - http://bmjopen.bmj.com/site/about/guidelines.xhtml

Variable	Total	Newborn separation: Not separated	Newborn separation: Separated	p-value	Newborn separation >10min: Not separated or separated ≤10min	Newborn separation >10min: Separated >10min	p-value	Consent sought for newborn procedures[1]: Did not ask for permission	Consent sought for newborn procedures[1]: Asked for permission	p-value
SD	(1.37)	(1.35)	(1.45)		(1.36)	(1.40)		(1.38)	(1.17)	
Breastfeeding care index										
Mean	2.00	2.04	1.80	0.006	2.01	1.88	0.173	1.79	2.30	<0.001
SD	(1.07)	(1.05)	(1.13)		(1.06)	(1.10)		(1.10)	(0.94)	

[1] Sample size, N=1,013 (1 refused to answer)
[2] For analyses, one missing response for newborn complications was recoded as "healthy, no issues."

The distributions of outcomes by reports of newborn separation, separation for more than 10 minutes, and consent sought for newborn procedures are presented in Table 4. Women who were asked permission for newborn procedures were more likely to be satisfied with care (p<0.001) and attend a postpartum visit after discharge (p<0.001) than those who were not asked permission.

Table 4. Distributions of outcomes by reports of newborn separation, separation for more than 10 minutes, and consent sought for newborn procedures (n=1,014)

Outcomes	Total	Newborn separated: Not separated	Newborn separated: Separated	p-value	Newborn separated >10min: Not separated or separated ≤ 10min	Newborn separated >10min: Separated >10min	p-value	Consent sought for newborn procedures[1]: Did not ask for permission	Consent sought for newborn procedures[1]: Asked for permission	p-value
Total number in group	1014	836	178		886	128		607	406	
Satisfaction with newborn care										
Dissatisfied	5.9%	6.1%	5.1%	0.592	6.1%	4.7%	0.528	8.4%	2.0%	<0.001
Satisfied	94.1%	93.9%	94.9%		93.9%	95.3%		91.6%	98.0%	
Attended postpartum visit after discharge, within 10 weeks										
No, did not attend	53.1%	53.5%	51.1%	0.569	53.4%	50.8%	0.581	57.7%	46.1%	<0.001
Yes, attended	46.9%	46.5%	48.9%		46.6%	49.2%		42.3%	53.9%	
Exclusively breastfeeding at 2-4 week follow-up [2]										
No	9.0%	8.5%	11.9%	0.193	8.5%	13.%	0.128	9.8%	8.0%	0.373
Yes	91.0%	91.5%	88.1%		91.5%	87.%		90.2%	92.0%	
Exclusively breastfeeding at 10 week follow-up [3]										

For peer review only - http://bmjopen.bmj.com/site/about/guidelines.xhtml

No	18.0%	17.7%	19.6%	0.577	17.6%	21.0%	0.399	20.1%	15.0%	0.058
Yes	82.0%	82.3%	80.4%		82.4%	79.0%		79.9%	85.0%	

[1] Sample size, N=1,013 (1 refused to answer)
[2] Sample size, N=829 (women who completed 2-4 week follow-up and reported on exclusive breastfeeding)
[3] Sample size, N=839 (women who completed 10 week follow-up and reported on exclusive breastfeeding)

Results of multivariate analyses examining associations between reports of newborn separation, consent sought for newborn procedures, and health outcomes are presented in Table 5. Among women reporting any newborn separation during care, there was a negative to no association with exclusive breastfeeding at 2-4 weeks (aOR=0.67, 95%CI: 0.41, 1.09). However, women who reported separation from their newborn longer than 10 minutes were 44% less likely to be exclusively breastfeeding at 2-4 weeks compared to those reporting less than 10 minutes or no separation (aOR=0.56, 95%CI: 0.40, 0.76). No associations were found with satisfaction with newborn care, postpartum visit attendance, or exclusive breastfeeding at 10 weeks.

Consent sought for newborn procedures was positively associated with satisfaction with newborn care at baseline, attending a postpartum visit by 10 weeks, and exclusive breastfeeding at 10 weeks. Women whose providers asked for permission before performing newborns' procedures or examinations were 2.7 times more likely to be satisfied with care (aOR=2.71, 95%CI: 1.67, 4.41) and were also 27% more likely to attend a postpartum visit by either 2-4 weeks or 10 weeks (aOR=1.27, 95%CI: 1.05, 4.41) after controlling for covariates. Consent for newborn procedures displayed no association with exclusive breastfeeding at 2-4 weeks (aOR=1.06, 95%CI: 0.78, 1.44), but was associated with a 33% increased likelihood of exclusive breastfeeding at 10 weeks (aOR=1.33, 95%CI: 1.10, 1.62) even after controlling for breastfeeding care index, and other previously described covariates.

For peer review only

Table 5. Results of multivariate logistic regression analyses examining the associations between separation, consent for newborn care, and maternal and newborn outcomes (n=1,014)

	Newborn separated from mother	Newborn separated for more than 10 minutes	Consent sought for newborn procedures [1]
**Outcome**	aOR (95% CI)	aOR (95% CI)	aOR (95% CI)
Satisfaction with newborn care at baseline *(ref. not satisfied)*	1.34 (0.75, 2.41)	1.36 (0.57, 3.28)	2.71 (1.67, 4.41)***
Attended a post-partum visit, within 10 weeks *(ref. no visit)*	1.14 (0.96, 1.35)	1.11 (0.86, 1.43)	1.27 (1.05, 1.55)*
Exclusively breastfeeding at 2-4 weeks [2] *(ref. not exclusively breastfed)*	0.67 (0.41, 1.09)	0.56 (0.40, 0.76)**	1.06 (0.78, 1.44) [3]
Exclusively breastfeeding at 10 weeks [4] *(ref. not exclusively breastfed)*	0.76 (0.49, 1.18)	0.66 (0.39, 1.10)	1.33 (1.10, 1.62)**

Note: All estimates of odds ratios (and 95% confidence intervals) were adjusted for age, multiparity, marital status, education, employment, women's birthplace, facility type, insurance status, health status, and clinical quality of care index. Satisfaction with newborn care models also adjusted for maternal complications and newborn complications at baseline. Breastfeeding outcome models also adjusted for breastfeeding care index, newborn complications at baseline, and both newborn and maternal complications after discharge. Post-partum visit models also adjusted for maternal and newborn complications after discharge. Cluster robust standard errors were used to account for clustering by facility.

[1] Sample size, N=1,013 (women who reported consent sought for newborn procedures, excluded 1 missing response)

[2] Sample size, N=829 (women who completed 2-4 week follow up survey and reported exclusive breastfeeding status)

[3] Sample size, N=828 (women who completed 2-4 week follow up survey and reported exclusive breastfeeding status, excluded 1 missing response for consent sought for newborn procedures)

[4] Sample size, N=839 (women who completed 10 week follow up survey and reported exclusive breastfeeding status)

*p<0.05, **p<0.01, ***p<0.001

DISCUSSION

The immediate postpartum period is a critical clinical period with high mortality risk, and an important social period for maternal-newborn bonding. Whether positive or negative, most

For peer review only - http://bmjopen.bmj.com/site/about/guidelines.xhtml

families will hold strong memories of experiences in this time, and it may impact their opinions about and willingness to seek care in the future, health practices in the home, and long-term health outcomes. We sought to examine two newborn care practices related to the experience of care, to understand the context of these practices, and to assess associations with relevant outcomes: care satisfaction, use and breastfeeding. This study provides evidence that treatment of newborns is an important component of quality and is likely to influence newborn health and postpartum practices.

Importantly, this study found significant inequities related to treatment of women and their newborns. For example, being consented for newborn care was correlated with insurance coverage, indicating that poorer women and their newborns may not be treated equally by the health system with regard to their clinical care. Women with higher parity, born outside of Nairobi or Kiambu counties, and experienced delivery complications were more likely to experience separation from their newborns. Mothers and newborns were most likely to be separated at government hospitals, where the most women in Kenya deliver. Although less common, separation did occur in smaller health centres and dispensaries as well as private facilities. Other studies have found that while larger hospitals provide better clinical quality care than smaller hospitals, women are also more likely to experience poor patient-centered care.[54] Further research is needed to understand how much of separation is driven by facility policies rather than individual provider behaviour. Infrastructural issues like lack of space and insufficient infection control procedures may influence policies at health facilities to limit family member entry or separate newborns.[55] In underfunded facilities, fatigue and stress of health workers likely influences their ability to provide high-quality care.[56,57] On the other hand,

For peer review only - http://bmjopen.bmj.com/site/about/guidelines.xhtml

providers may have been trained to promote practices that are no longer evidenced-based, such as misconceptions around separation for infection control.[58] Although drivers of poor quality and disrespectful care are not fully understood,[47] these findings suggest that they are likely multi-faceted and interactive. More research is needed in a wider range of settings and countries, to explore the potential contextual factors that might influence policies, behaviours, and solutions.

In this study, 17.6% of women reported being separated from their newborns, which is less than has been reported in other studies.[48] Even so, many of these occurrences were thought by women to be unnecessary, and almost 10% were not given a reason for separation. Most separations were over 10 minutes and in only one-fifth was another family member able to accompany the newborn. In many contexts, and Kenya, specifically, fathers have limited access to maternity and postnatal wards and neonatal intensive care units.[8] Notably, other studies have found that women in Nairobi were less likely to want a companion during labor and delivery compared to women in rural Kenya, Ghana, and India; and were also less likely to report being allowed a companion all the time.[8,12] However, there is a potentially important role for fathers, family members, or doulas, in staying with the newborn when mothers require rest, surgery, or critical care.[27] Further research is needed to examine facility-level policies in allowing a companion of choice and facilitating family support when it is desired.

Alarmingly, over 60% of women reported not being consented for newborn procedures. Being asked to provide consent was correlated with higher scores on the clinical quality of care and breastfeeding indices. This may reflect that providers or facilities that are more consistent in the

For peer review only - http://bmjopen.bmj.com/site/about/guidelines.xhtml

provision of high quality care are also more likely to seek permission for procedures. This is consistent with another study in Kenya that found high levels of failure to provide informed consent and notable patient-provider discordance in reports of informed consent. Even in instances where providers reported obtaining consent from women for medical procedures, women were not provided full information or comprehend the information given to them.[14] We found that for both satisfaction and postpartum care utilization, being asked for permission for newborn care procedures was positively associated, further illustrating the importance of consent as an aspect of respectful care.

This study provides evidence that newborn treatment during childbirth is associated with newborn health and postpartum practices. Separation of newborns from their mothers for over 10 minutes was associated with decreased breastfeeding at a later time point. While this association could be confounded by newborns with more severe complications–which require additional procedures, and also affect breastfeeding–the association remained after adjustment in the model. It is also plausible that the lack of early initiation of breastfeeding and lower satisfaction with immediate postnatal care contributed to poorer breastfeeding practices after discharge because of lower motivation or encouragement. In addition to improved satisfaction and likelihood to utilize postnatal care, consent for newborn procedures was associated with a greater likelihood of exclusive breastfeeding at follow-up, indicating a potential causal link. This finding is important given the overwhelming evidence of the benefits of breastmilk for newborn survival, development and health of infants.[59,60] This adds to the evidence found by other studies which showed that lack of respectful and supportive care during intrapartum care is associated with more reports of newborn complications.[52]

For peer review only - http://bmjopen.bmj.com/site/about/guidelines.xhtml

Strengths and limitations

This study is one of the first studies to include questions with the primary aim of understanding details around newborn separation, and to correlate newborn care practices with subsequent health outcomes. While previous studies have reported on prevalence of newborn separation,[7,10,47] this study is the first to differentiate separation by maternal perception of reason and need, highlighting both the probability that much of the separation is not medically necessary, and that many parents do not fully understand the reasons for separation most likely due to poor communication. This study also goes beyond measuring maternal consent for care of self to consent of procedures for the newborn. Lastly, this study used longitudinal data and was able to measure treatment of newborns from pre-discharge care in facilities to postpartum practices.

This study was limited to 10 weeks of follow up, and it is unknown how care practices or experience of care for newborns may influence later pediatric care. Additionally, the samples for the 2-4 and 10 week follow-ups differed slightly (though not significantly across socio-demographics or care), so our comparisons of exclusive breastfeeding between these time periods may be interpreted conservatively due to smaller sample size and potential biases due to composition. Further, this study focused on women's reports of their care and did not triangulate with hospital records or other sources of information; thus, participants may have had recall bias, may have defined "separation" differently than the researchers, and may have different interpretations than health care providers about certain practices, such as necessity of separation. While it was explained to participants that this study was confidential and was being conducted

For peer review only - http://bmjopen.bmj.com/site/about/guidelines.xhtml

independently of the facility or health system, there may have been desirability bias if respondents were concerned about their subsequent care at facilities. Lastly, our results may have limited generalizability, as our study sample included facilities from Nairobi and Kiambu Counties, Kenya. Future research may explore whether associations with these care practices are also present in other social and institutional contexts.

Conclusions

Newborns, their mothers, and their families have a right to high quality, respectful care, regardless of their characteristics or where they are delivered. High quality newborn care includes the ability to stay together and for the parents to be informed and consent for care. The implications of various practices on health outcomes a month or more after discharge illustrate the importance of a positive experience of postnatal care. More research and action are needed to provide newborns with the standard of care that they deserve.

Contributors

MS and ES conceptualized and designed the study. MN analyzed the data. MN, ES, JO, and MS contributed to the writing and revision of the manuscript.

Acknowledgements

We would like to thank Ginger Golub and Sun Cotter for assistance in collection of data. We also express gratitude and appreciation to all of the women who participated in this study.

Data Sharing

For peer review only

Data are available upon reasonable request. Please contact the corresponding author with requests for de-identified participant data.

Funding statement

This study was funded by the Bill and Melinda Gates Foundation. The authors also thank the California Center for Population Research at UCLA (CCPR) for its training support from the Eunice Kennedy Shriver National Institute of Child Health and Human Development (T32-HD007545).

Competing interests

None declared.

For peer review only

References

1. UN IGME. Levels & Trends in Child Mortality: Report 2019, Estimates developed by the UN Inter-agency Group for Child Mortality Estimation. New York; 2019.
2. HNN. Kenya Data [Internet]. Healthy Newborn Network. 2020 [cited 2020 Aug 6]. Available from: https://www.healthynewbornnetwork.org/country/kenya/
3. UN IGME. Kenya Neonatal Mortality Estimates [Internet]. UN IGME. 2018 [cited 2020 Aug 6]. Available from: https://childmortality.org/data/Kenya
4. WHO, UNICEF. Every Newborn: an action plan to end preventable deaths [Internet]. Geneva; 2014.
5. Tunçalp, Were WM, Maclennan C, Oladapo OT, Gülmezoglu AM, Bahl R, et al. Quality of care for pregnant women and newborns - The WHO vision. BJOG An Int J Obstet Gynaecol. 2015 Jul 1;122(8):1045–9.
6. Rosen HE, Lynam PF, Carr C, Reis V, Ricca J, Bazant ES, et al. Direct observation of respectful maternity care in five countries: a cross-sectional study of health facilities in East and Southern Africa. BMC Pregnancy Childbirth [Internet]. 2015 Dec 23;15(1):306.
7. Reis V, Deller B, Carr C, Smith J. Respectful maternity care. Country experience [Internet]. Alliance francophone pour l'accouchement respecté (AFAR); 2016.
8. Afulani PA, Phillips B, Aborigo RA, Moyer CA. Person-centred maternity care in low-income and middle-income countries: analysis of data from Kenya, Ghana, and India. Lancet Glob Heal. 2019 Jan 1;7(1):e96–109.
9. McMahon SA, George AS, Chebet JJ, Mosha IH, Mpembeni RN, Winch PJ. Experiences of and responses to disrespectful maternity care and abuse during childbirth; a qualitative study with women and men in Morogoro Region, Tanzania. BMC Pregnancy Childbirth [Internet]. 2014 Dec 12;14(1):268.
10. Bohren MA, Mehrtash H, Fawole B, Maung TM, Balde MD, Maya E, et al. How women are treated during facility-based childbirth in four countries: a cross-sectional study with labour observations and community-based surveys. Lancet. 2019 Nov 9;394(10210):1750–63.
11. Bohren MA, Hunter EC, Munthe-Kaas HM, Souza JP, Vogel JP, Gülmezoglu AM. Facilitators and barriers to facility-based delivery in low- and middle-income countries: A qualitative evidence synthesis. Vol. 11, Reproductive Health. BioMed Central Ltd.; 2014.
12. Afulani P, Kusi C, Kirumbi L, Walker D. Companionship during facility-based childbirth: results from a mixed-methods study with recently delivered women and providers in Kenya. BMC Pregnancy Childbirth [Internet]. 2018 Dec 10;18(1):150.
13. Kabakian-Khasholian T, Portela A. Companion of choice at birth: Factors affecting implementation. BMC Pregnancy Childbirth [Internet]. 2017 Aug 31;17(1):265.
14. Sudhinaraset M, Giessler K, Golub G, Afulani P. Providers and women's perspectives on person-centered maternity care: A mixed methods study in Kenya [Internet]. Vol. 18, International Journal for Equity in Health. BioMed Central Ltd.; 2019. p. 83.
15. Afulani PA, Kirumbi L, Lyndon A. What makes or mars the facility-based childbirth experience: Thematic analysis of women's childbirth experiences in western Kenya. Reprod Health [Internet]. 2017 Dec 29;14(1):180.
16. Downe S, Finlayson K, Oladapo O, Bonet M, Gülmezoglu AM. What matters to women during childbirth: A systematic qualitative review [Internet]. Vol. 13, PLoS ONE. Public Library of Science; 2018. p. e0194906.
17. Brüggemann OM, Osis MJD, Parpinelli MA. Support during childbirth: Perception of

For peer review only - http://bmjopen.bmj.com/site/about/guidelines.xhtml

health care providers and companions chosen by women. Rev Saude Publica. 2007;41(1):1–9.
18. Owiti A, Oyugi J, Essink D. Utilization of Kenya's free maternal health services among women living in Kibera slums: A cross-sectional study. Pan Afr Med J. 2018;30.
19. Escamilla V, Calhoun L, Winston J, Speizer IS. The Role of Distance and Quality on Facility Selection for Maternal and Child Health Services in Urban Kenya. J Urban Heal. 2018 Feb 1;95(1).
20. Keats EC, Ngugi A, Macharia W, Akseer N, Khaemba EN, Bhatti Z, et al. Progress and priorities for reproductive, maternal, newborn, and child health in Kenya: a Countdown to 2015 country case study. Lancet Glob Heal. 2017 Aug 1;5(8):e782–95.
21. Sharma J, Leslie HH, Kundu F, Kruk ME. Poor quality for poor women? Inequities in the quality of antenatal and delivery care in Kenya. PLoS One. 2017 Jan 1;12(1).
22. Kruk ME, Pate M. The Lancet Global Health Commission on High Quality Health Systems 1 year on: progress on a global imperative [Internet]. Vol. 8, The Lancet Global Health. Elsevier Ltd; 2020. p. e30–2.
23. WHO. WHO standards of care to improve maternal and newborn quality of care in facilities [Internet]. Geneva; 2016.
24. Sacks E, Kinney M V. Respectful maternal and newborn care: Building a common agenda [Internet]. Vol. 12, Reproductive Health. BioMed Central Ltd.; 2015.
25. Klaus MH. Commentary: An Early, Short, and Useful Sensitive Period in the Human Infant. Birth [Internet]. 2009 Jun 1;36(2):110–2.
26. Sullivan R, Perry R, Sloan A, Kleinhaus K, Burtchen N. Infant Bonding and Attachment to the Caregiver: Insights from Basic and Clinical Science [Internet]. Vol. 38, Clinics in Perinatology. NIH Public Access; 2011. p. 643–55.
27. Widström A, Brimdyr K, Svensson K, Cadwell K, Nissen E. Skin-to-skin contact the first hour after birth, underlying implications and clinical practice. Acta Paediatr [Internet]. 2019 Jul 13;108(7):1192–204.
28. Bergman N. Birth practices: Maternal-neonate separation as a source of toxic stress [Internet]. Vol. 111, Birth Defects Research. John Wiley and Sons Inc.; 2019. p. 1087–109.
29. Hofer MA. Psychobiological Roots of Early Attachment. Curr Dir Psychol Sci [Internet]. 2006 Apr 22;15(2):84–8.
30. Stevens J, Schmied V, Burns E, Dahlen H. Immediate or early skin-to-skin contact after a Caesarean section: A review of the literature [Internet]. Vol. 10, Maternal and Child Nutrition. Blackwell Publishing Ltd; 2014. p. 456–73.
31. Ludington-Hoe SM, Swinth JY. Developmental aspects of kangaroo care. J Obstet Gynecol Neonatal Nurs [Internet]. 1996;25(8):691–703.
32. Feldman R, Eidelman AI. Skin-to-skin contact (Kangaroo Care) accelerates autonomic and neurobehavioural maturation in preterm infants. Dev Med Child Neurol. 2003 Apr 1;45(4):274–81.
33. Loman MM, Bruce J, Dallman M, Dozier M, Fisher P, Fox N, et al. Early Experience and the Development of Stress Reactivity and Regulation in Children Gunnar [on behalf of the Early Experience, Stress, and Neurobehavioral Development Center. Neurosci Biobehav Rev. 2010;34(6):867–76.
34. Champagne F, Meaney MJ. Like mother, like daughter: Evidence for non-genomic transmission of parental behavior and stress responsivity. In: Progress in Brain Research.

For peer review only

For peer review only - http://bmjopen.bmj.com/site/about/guidelines.xhtml

Elsevier; 2001. p. 287–302.
35. Shonkoff JP, Garner AS, Siegel BS, Dobbins MI, Earls MF, McGuinn L, et al. The lifelong effects of early childhood adversity and toxic stress. Pediatrics [Internet]. 2012 Jan;129(1).
36. Bystrova K, Ivanova V, Edhborg M, Matthiesen AS, Ransjö-Arvidson AB, Mukhamedrakhimov R, et al. Early contact versus separation: Effects on mother-infant interaction one year later. Birth [Internet]. 2009 Jun;36(2):97–109.
37. Swain JE, Lorberbaum JP, Kose S, Strathearn L. Brain basis of early parent-infant interactions: Psychology, physiology, and in vivo functional neuroimaging studies. J Child Psychol Psychiatry Allied Discip [Internet]. 2007 Mar;48(3–4):262–87.
38. Bigelow A, Power M, Maclellan-Peters J, Alex M, Mcdonald C. Effect of Mother/Infant Skin-to-Skin Contact on Postpartum Depressive Symptoms and Maternal Physiological Stress. JOGNN - J Obstet Gynecol Neonatal Nurs [Internet]. 2012;41(3):369–82.
39. Strathearn L, Mamun AA, Najman JM, O'Callaghan MJ. Does breastfeeding protect against substantiated child abuse and neglect? A 15-year cohort study. Pediatrics [Internet]. 2009 Feb;123(2):483–93.
40. Scism AR, Cobb RL. Integrative Review of Factors and Interventions That Influence Early Father–Infant Bonding. Vol. 46, JOGNN - Journal of Obstetric, Gynecologic, and Neonatal Nursing. Elsevier B.V.; 2017. p. 163–70.
41. Hallowell SG, Froh EB, Spatz DL. Human milk and breastfeeding: An intervention to mitigate toxic stress. Nurs Outlook [Internet]. 2017 Jan 1;65(1):58–67.
42. Uvnäs-Moberg K. Neuroendocrinology of the mother-child interaction [Internet]. Vol. 7, Trends in Endocrinology and Metabolism. Elsevier Inc.; 1996. p. 126–31.
43. Moore ER, Bergman N, Anderson GC, Medley N. Early skin-to-skin contact for mothers and their healthy newborn infants [Internet]. Vol. 2016, Cochrane Database of Systematic Reviews. John Wiley and Sons Ltd; 2016.
44. Koster D, Romijn C, Sakko E, Stam C, Steenhuis N, de Vries D, et al. Traumatic childbirth experiences: practice-based implications for maternity care professionals from the woman's perspective. Scand J Caring Sci [Internet]. 2019;
45. Jolly Y, Aminu M, Mgawadere F, Van Den Broek N. "We are the ones who should make the decision" - Knowledge and understanding of the rights-based approach to maternity care among women and healthcare providers. BMC Pregnancy Childbirth [Internet]. 2019 Feb 15;19(1).
46. Shakibazadeh E, Namadian M, Bohren MA, Vogel JP, Rashidian A, Nogueira Pileggi V, et al. Respectful care during childbirth in health facilities globally: a qualitative evidence synthesis [Internet]. Vol. 125, BJOG: An International Journal of Obstetrics and Gynaecology. Blackwell Publishing Ltd; 2018. p. 932–42.
47. Sacks E. Defining disrespect and abuse of newborns: A review of the evidence and an expanded typology of respectful maternity care [Internet]. Vol. 14, Reproductive Health. BioMed Central Ltd.; 2017. p. 66.
48. Sacks E, Mehrtash H, Bohren MA, Balde MD, Vogel JP, Adu-Bonsaffoh K, et al. The first two hours after birth: prevalence and factors associated with newborn care practices from a multi-country, facility-based observational study. Lancet Glob Heal. 2020;in press.
49. Waiswa P, Nyanzi S, Namusoko-Kalungi S, Peterson S, Tomson G, Pariyo GW. 'I never thought that this baby would survive; I thought that it would die any time': perceptions and care for preterm babies in eastern Uganda. Trop Med Int Heal [Internet]. 2010 Oct

For peer review only - http://bmjopen.bmj.com/site/about/guidelines.xhtml

1;15(10):1140–7.
50. Tolhurst R, Theobald S, Kayira E, Ntonya C, Kafulafula G, Nielson J, et al. “I don’t want all my babies to go to the grave”: perceptions of preterm birth in Southern Malawi. Midwifery. 2008 Mar 1;24(1):83–98.
51. Crossley MA. Of diagnoses and discrimination: discriminatory nontreatment of infants with HIV infection. Columbia Law Rev. 1993;93(7):1581–667.
52. Sudhinaraset M, Landrian A, Afulani PA, Diamond-Smith N, Golub G. Association between person-centered maternity care and newborn complications in Kenya. Int J Gynecol Obstet [Internet]. 2020 Jan 9;148(1):27–34.
53. Strathearn L. Maternal neglect: Oxytocin, dopamine and the neurobiology of attachment [Internet]. Vol. 23, Journal of Neuroendocrinology. J Neuroendocrinol; 2011. p. 1054–65.
54. Phillips B, Kajal F, Montagu D, Kumar A, Kumar V. Is bigger better? Assessment of self-reported and researcher-collected data on maternal health care quality among high-case-load facilities in Uttar Pradesh: a mixed-methods study. Lancet Glob Heal [Internet]. 2018 Mar 1;6:S46.
55. Sacks E, Sripad P, Ndwiga C, Waiswa P, Warren CE. Protecting newborn infants during the COVID-19 pandemic should be based on evidence and equity. Acta Paediatr [Internet]. 2020;
56. Mgawadere F, Smith H, Asfaw A, Lambert J, Broek N van den. “There is no time for knowing each other”: Quality of care during childbirth in a low resource setting. Midwifery [Internet]. 2019 Aug 1;75:33–40.
57. O’Donnell E, Utz B, Khonje D, van den Broek N. “At the right time, in the right way, with the right resources”: Perceptions of the quality of care provided during childbirth in Malawi. BMC Pregnancy Childbirth [Internet]. 2014 Jul 28;14(1):248.
58. Maimbolwa MC, Sikazwe N, Yamba B, Diwan V, Ransjö-Arvidson AB. Views on involving a social support person during labor in Zambian maternities. J Midwifery Womens Health [Internet]. 2001;46(4):226–34.
59. Lyons KE, Ryan CA, Dempsey EM, Ross RP, Stanton C. Breast Milk, a Source of Beneficial Microbes and Associated Benefits for Infant Health. Nutrients [Internet]. 2020 Apr 9;12(4):1039.
60. Paduraru D. The evidence for the benefits from breast milk in the neurodevelopment of premature babies – a review of the recent literature. J Mind Med Sci [Internet]. 2018 Oct 3;5(2):151–7.

For peer review only

Supplement 1. Flowchart of baseline and follow-up analytic sample sizes

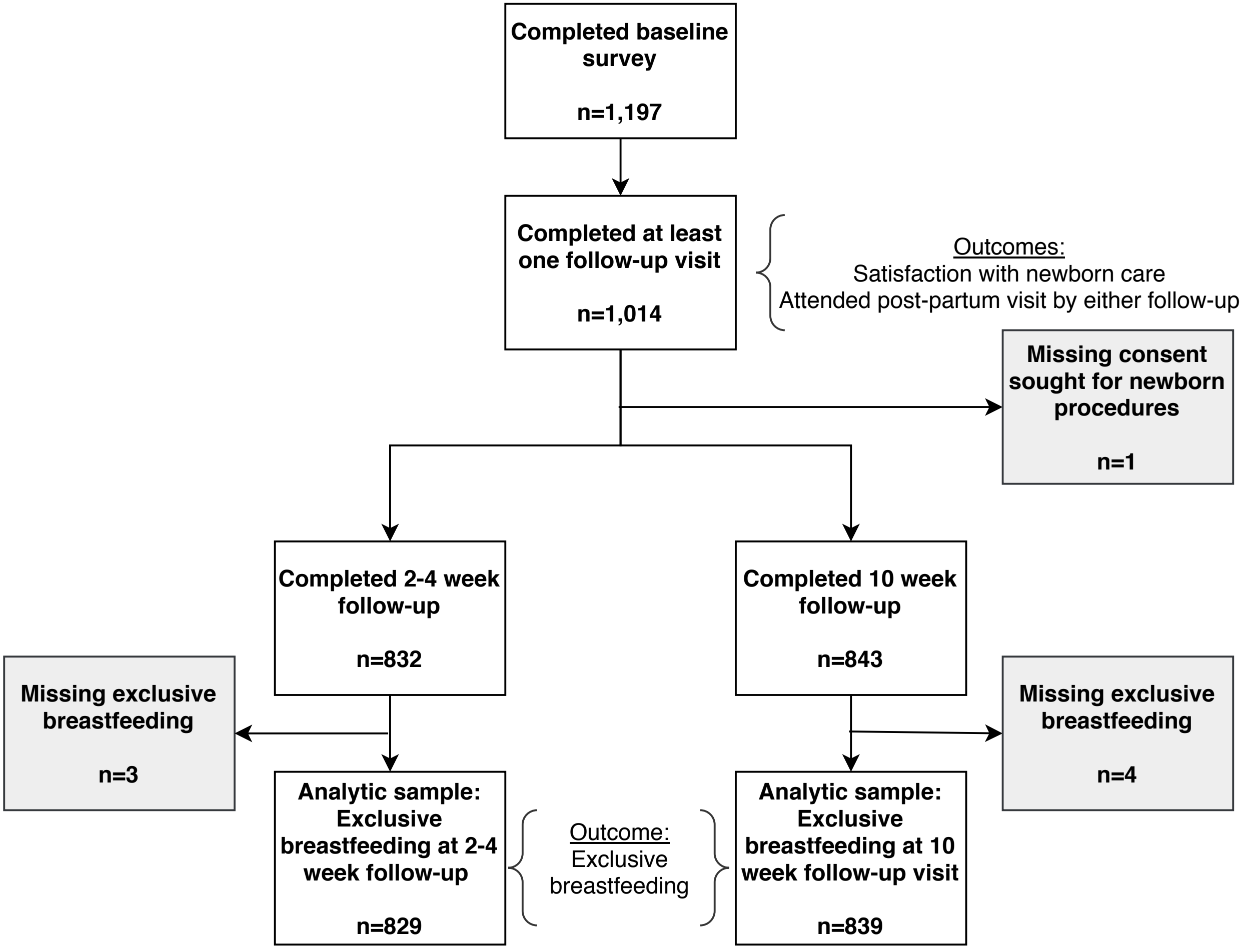

Supplement 2. Distributions of clinical and breastfeeding care indicators by reports of newborn separation and newborn consent indicators, (n=1,014)

Variable	Total	Newborn separation: Not separated	Newborn separation: Separated	Newborn separation: p-value	Newborn separation >10min: Not separated or separated ≤ 10min	Newborn separation >10min: Separated >10min	Newborn separation >10min: p-value	Consent sought for newborn procedures[1]: Did not ask for permission	Consent sought for newborn procedures[1]: Asked for permission	Consent sought for newborn procedures[1]: p-value
Total number in group	1,014	836	178		886	128		607	406	
**Clinical quality of care indicators**										
Was your baby put on your abdomen or chest as soon as it was born?										
No	13.6%	13.0%	16.3%	0.467	13.0%	18.0%	0.287	15.8%	10.3%	0.032
Yes	86.3%	86.8%	83.7%		86.9%	82.0%		84.0%	89.7%	
Don't know	0.1%	0.1%	0.0%		0.1%	0.0%		0.2%	0.0%	
Within two hours of delivery, did a health provider examine your baby?										
No	25.0%	25.7%	21.9%	0.049	26.4%	15.6%	0.001	31.3%	15.5%	<0.001
Yes	74.1%	73.7%	75.8%		73.0%	81.2%		67.5%	84.%	
Don't know	0.8%	0.6%	1.7%		0.6%	2.3%		1.2%	0.2%	
Missing	0.1%	0.0%	0.6%		0.0%	0.8%		0.0%	0.2%	
Was your baby wiped dry within a few minutes after birth?										
No	7.4%	7.3%	7.9%	0.013	7.2%	8.6%	0.091	9.2%	4.7%	0.003
Yes	90.4%	91.1%	87.1%		91.0%	86.7%		88.0%	94.3%	
Don't know	2.2%	1.6%	5.1%		1.8%	4.7%		2.8%	1.0%	
Has your baby been bathed yet?										
No	92.6%	93.3%	89.3%	<0.00	93.3%	87.5%	<0.00	92.6%	92.6%	0.723
Yes	5.8%	5.9%	5.6%		5.9%	5.5%		5.6%	6.2%	
Don't know	1.6%	0.8%	5.1%		0.8%	7.0%		1.8%	1.2%	
After birth, did any health care provider examine the cord?										
No	28.0%	27.4%	30.9%	0.344	27.9%	28.9%	0.809	34.8%	18.0%	<0.001
Yes	72.0%	72.6%	69.1%		72.1%	71.1%		65.2%	82.0%	
After birth, did any health care provider assess the temperature of your baby?										
No	51.1%	52.6%	43.8%	0.033	52.0%	44.5%	0.113	56.3%	43.1%	<0.001
Yes	48.9%	47.4%	56.2%		48.0%	55.5%		43.7%	56.9%	
After birth, did any health care provider counsel you on danger signs for newborns?										
No	63.4%	63.4%	63.5%	0.983	63.4%	63.3%	0.974	73.0%	49.0%	<0.001
Yes	36.6%	36.6%	36.5%		36.6%	36.7%		27.0%	51.0%	
**Breastfeeding care indicators**										
Within two hours of delivery, did a health provider check if breastfeeding was going well.										
No	24.2%	22.8%	30.3%	0.048	23.9%	25.8%	0.248	29.8%	15.8%	<0.001
Yes	75.6%	77.0%	69.1%		76.0%	73.4%		69.9%	84.2%	
Don't know	0.2%	0.1%	0.6%		0.1%	0.8%		0.3%	0.0%	
While you were in the hospital for the delivery of this baby did any one counsel/talk to you about breastfeeding?										
No	34.6%	33.5%	39.9%	0.103	34.%	39.1%	0.258	40.7%	25.4%	<0.001
Yes	65.4%	66.5%	60.1%		66.%	60.9%		59.3%	74.6%	
While you were in the hospital for the delivery of this baby did any one help you with breastfeeding by observing or showing you how to breastfeed?										
No	42.4%	40.6%	50.9%	0.013	41.5%	48.8%	0.126	51.1%	29.6%	<0.001
Yes	57.6%	59.4%	49.1%		58.5%	51.2%		48.9%	70.4%	

Note: For analyses, clinical quality and breastfeeding care indicators, "don't know" responses were recoded as "no," and one missing response was conservatively recoded as "yes."

For peer review only - http://bmjopen.bmj.com/site/about/guidelines.xhtml

STROBE Statement—Checklist of items that should be included in reports of ***cohort studies***

	Item No	Recommendation	Page No
**Title and abstract**	1	(*a*) Indicate the study's design with a commonly used term in the title or the abstract	1,2
		(*b*) Provide in the abstract an informative and balanced summary of what was done and what was found	2
**Introduction**			
Background/rationale	2	Explain the scientific background and rationale for the investigation being reported	3-7
Objectives	3	State specific objectives, including any prespecified hypotheses	7
**Methods**			
Study design	4	Present key elements of study design early in the paper	8
Setting	5	Describe the setting, locations, and relevant dates, including periods of recruitment, exposure, follow-up, and data collection	8-9
Participants	6	(*a*) Give the eligibility criteria, and the sources and methods of selection of participants. Describe methods of follow-up<br>(*b*) For matched studies, give matching criteria and number of exposed and unexposed	8-9
Variables	7	Clearly define all outcomes, exposures, predictors, potential confounders, and effect modifiers. Give diagnostic criteria, if applicable	9-11
Data sources/ measurement	8*	For each variable of interest, give sources of data and details of methods of assessment (measurement). Describe comparability of assessment methods if there is more than one group	9-11
Bias	9	Describe any efforts to address potential sources of bias	10-11
Study size	10	Explain how the study size was arrived at	12, Supp 1
Quantitative variables	11	Explain how quantitative variables were handled in the analyses. If applicable, describe which groupings were chosen and why	9-10
Statistical methods	12	(*a*) Describe all statistical methods, including those used to control for confounding<br>(*b*) Describe any methods used to examine subgroups and interactions<br>(*c*) Explain how missing data were addressed<br>(*d*) If applicable, explain how loss to follow-up was addressed<br>(*e*) Describe any sensitivity analyses	10-11, Table footnotes, Supplement 1
**Results**			
Participants	13*	(a) Report numbers of individuals at each stage of study—eg numbers potentially eligible, examined for eligibility, confirmed eligible, included in the study, completing follow-up, and analysed<br>(b) Give reasons for non-participation at each stage<br>(c) Consider use of a flow diagram	9, Supp 1
Descriptive data	14*	(a) Give characteristics of study participants (eg demographic, clinical, social) and information on exposures and potential confounders<br>(b) Indicate number of participants with missing data for each variable of interest<br>(c) Summarise follow-up time (eg, average and total amount)	12-15, Table footnotes
Outcome data	15*	Report numbers of outcome events or summary measures over time	16-17

For peer review only

For peer review only - http://bmjopen.bmj.com/site/about/guidelines.xhtml

Main results	16	(*a*) Give unadjusted estimates and, if applicable, confounder-adjusted estimates and their precision (eg, 95% confidence interval). Make clear which confounders were adjusted for and why they were included<br>(*b*) Report category boundaries when continuous variables were categorized<br>(*c*) If relevant, consider translating estimates of relative risk into absolute risk for a meaningful time period	16-18
Other analyses	17	Report other analyses done—eg analyses of subgroups and interactions, and sensitivity analyses	11
**Discussion**			
Key results	18	Summarise key results with reference to study objectives	18-21
Limitations	19	Discuss limitations of the study, taking into account sources of potential bias or imprecision. Discuss both direction and magnitude of any potential bias	22-23
Interpretation	20	Give a cautious overall interpretation of results considering objectives, limitations, multiplicity of analyses, results from similar studies, and other relevant evidence	18-21
Generalisability	21	Discuss the generalisability (external validity) of the study results	23
**Other information**			
Funding	22	Give the source of funding and the role of the funders for the present study and, if applicable, for the original study on which the present article is based	24

*Give information separately for exposed and unexposed groups.

Note: An Explanation and Elaboration article discusses each checklist item and gives methodological background and published examples of transparent reporting. The STROBE checklist is best used in conjunction with this article (freely available on the Web sites of PLoS Medicine at http://www.plosmedicine.org/, Annals of Internal Medicine at http://www.annals.org/, and Epidemiology at http://www.epidem.com/). Information on the STROBE Initiative is available at http://www.strobe-statement.org.

For peer review only

For peer review only - http://bmjopen.bmj.com/site/about/guidelines.xhtml

BMJ Open

The Association between Newborn Separation, Maternal Consent, and Health Outcomes: Findings from a Longitudinal Survey in Kenya

Journal:	*BMJ Open*
Manuscript ID	bmjopen-2020-045907.R1
Article Type:	Original research
Date Submitted by the Author:	26-Apr-2021
Complete List of Authors:	Nakphong, Michelle; University of California Los Angeles, Community Health Sciences<br>Sacks, Emma; Johns Hopkins University Bloomberg School of Public Health, Department of International Health<br>Opot, James; Innovations for Poverty Action<br>Sudhinaraset, May; University of California Los Angeles, Department of Community Health Sciences
**Primary Subject Heading**:	Obstetrics and gynaecology
Secondary Subject Heading:	Health services research
Keywords:	OBSTETRICS, Health policy < HEALTH SERVICES ADMINISTRATION & MANAGEMENT, International health services < HEALTH SERVICES ADMINISTRATION & MANAGEMENT, Quality in health care < HEALTH SERVICES ADMINISTRATION & MANAGEMENT

SCHOLARONE™
Manuscripts

I, the Submitting Author has the right to grant and does grant on behalf of all authors of the Work (as defined in the below author licence), an exclusive licence and/or a non-exclusive licence for contributions from authors who are: i) UK Crown employees; ii) where BMJ has agreed a CC-BY licence shall apply, and/or iii) in accordance with the terms applicable for US Federal Government officers or employees acting as part of their official duties; on a worldwide, perpetual, irrevocable, royalty-free basis to BMJ Publishing Group Ltd ("BMJ") its licensees and where the relevant Journal is co-owned by BMJ to the co-owners of the Journal, to publish the Work in this journal and any other BMJ products and to exploit all rights, as set out in our licence.

The Submitting Author accepts and understands that any supply made under these terms is made by BMJ to the Submitting Author unless you are acting as an employee on behalf of your employer or a postgraduate student of an affiliated institution which is paying any applicable article publishing charge ("APC") for Open Access articles. Where the Submitting Author wishes to make the Work available on an Open Access basis (and intends to pay the relevant APC), the terms of reuse of such Open Access shall be governed by a Creative Commons licence – details of these licences and which Creative Commons licence will apply to this Work are set out in our licence referred to above.

Other than as permitted in any relevant BMJ Author's Self Archiving Policies, I confirm this Work has not been accepted for publication elsewhere, is not being considered for publication elsewhere and does not duplicate material already published. I confirm all authors consent to publication of this Work and authorise the granting of this licence.

For peer review only

The Association between Newborn Separation, Maternal Consent, and Health Outcomes: Findings from a longitudinal survey in Kenya

Michelle K. Nakphong[1]*, Emma Sacks[2], James Opot[3], May Sudhinaraset[1]

[1] Department of Community Health Sciences, University of California, Los Angeles, CA, USA
[2] Department of International Health, Johns Hopkins School of Public Health, Baltimore, MD, USA
[3] Innovations for Poverty Action, Nairobi, Kenya

***Corresponding author:** Department of Community Health Sciences, University of California, Los Angeles; 650 Charles E. Young Drive South, 36-071 CHS, Box 951772 Los Angeles, CA 90095-1772. Email: hmkao@ucla.edu.

Word count: 4378

For peer review only

The Association Between Newborn Separation, Maternal Consent, and Health Outcomes: Findings from a Longitudinal Survey in Kenya

Abstract

Objectives Disrespectful and poor treatment of newborns such as unnecessary separation from parents or failure to obtain parental consent for medical procedures occurs at health facilities across contexts, but little research has investigated the prevalence, risk factors, or associated outcomes. This study examined these experiences and associations with health care satisfaction, use, and breastfeeding.

Design Prospective cohort study

Setting 3 public hospitals, 2 private hospitals, and 1 health centre/dispensary in Nairobi and Kiambu counties in Kenya

Participants Data were collected from women who delivered in health facilities between September 2019 and January 2020. The sample included 1,014 women surveyed at baseline and at least one follow-up at 2-4 or 10 weeks postpartum.

Primary and secondary outcome measures 1) Outcomes related to satisfaction with care and care utilization, 2) Continuation of post-discharge newborn care practices such as breastfeeding.

Results 17.6% of women reported newborn separation at the facility, of whom 71.9% were separated over 10 minutes. 44.9% felt separation was unnecessary and 8.4% reported not knowing the reason for separation. 59.9% reported consent was not obtained for procedures on their newborn. Women separated from their newborn (>10 minutes) were 44% less likely to be exclusively breastfeeding at 2-4 weeks (aOR=0.56, 95%CI: 0.40, 0.76). Obtaining consent for newborn procedures corresponded with 2.7 times greater likelihood of satisfaction with care (aOR=2.71, 95%CI: 1.67, 4.41), 27% greater likelihood of postpartum visit attendance for self or newborn (aOR=1.27, 95%CI: 1.05, 4.41), and 33% greater likelihood of exclusive breastfeeding at 10 weeks (aOR=1.33, 95%CI: 1.10, 1.62).

Conclusions Newborns, mothers, and families have a right to high quality, respectful care, including the ability to stay together, be informed and proper consent for care. The implications of these experiences on health outcomes a month or more after discharge illustrate the importance of a positive experience of postnatal care.

Keywords

Newborn care; neonatal health; maternal child health; postnatal care; maternal-newborn separation; breastfeeding; respectful care; informed consent; quality of care; survey; observational data

For peer review only - http://bmjopen.bmj.com/site/about/guidelines.xhtml

Article Summary

Strengths and Limitations of the Study

- This is one of the first studies to include survey questions surrounding details of separation of newborns from mothers and maternal consent for care, including risk factors and reasons for separation.
- This study used longitudinal data collected over 10 weeks following delivery to assess associations with outcomes, including postpartum visit attendance and breastfeeding.
- Differences in participants who responded to the 2-4week and 10 week follow-up interviews may have introduced bias due to differences in composition, thus comparisons of associations with outcomes between these two points should be interpreted conservatively.
- Findings may have limited generalizability to other contexts, as the study sample included women who gave birth at facilities in Nairobi and Kiambu counties.

INTRODUCTION

The first month of life constitutes the most vulnerable time for a child's survival. Globally, 2.4 million children die within the first 28 days of life at a rate of 18 deaths per 1,000 live births.[1] Kenya has made notable progress in reducing neonatal mortality in recent years, [2] but with its current neonatal mortality ratio of 19.6 deaths per 1,000 live births, Kenya is unlikely to reach the 2030 Sustainable Development Goal target of 12 deaths per 1,000 live births.[3] Yet, substantial progress can still be made: over 80% of newborn deaths are considered preventable, highlighting the critical need to improve the quality of maternal and newborn care.[4]

Efforts to enhance the quality of maternity care have drawn attention to women's experience of care, including respect and dignity. The WHO Vision on Quality of Care for maternal and newborn health outlines two essential, interlinked dimensions of Quality of Care: provision of care and experience of care.[5] Integrally, quality care, in addition to being safe, effective,

For peer review only - http://bmjopen.bmj.com/site/about/guidelines.xhtml

timely, and efficient, must also be equitable and people-centered. Experience of care includes effective communication, respectful and dignified treatment, and access to emotional support. Evidence indicates that across contexts, many women experience mistreatment and abuse,[6–11] and lack access to the support person(s) of their choice during labor and delivery.[12–14] Negative experiences of care not only contribute to dissatisfaction with care,[15–17] but may deter women from seeking future health services.[18–22] However, the corresponding elements of quality newborn care and the health impact of these experiences have been under-researched.

WHO recommendations for newborn care include skin-to-skin care, early and exclusive breastfeeding, thermal and umbilical care, delayed bathing, and maintaining the newborn with family as much as possible.[23] These practices are especially important for preterm and low birth weight infants, who are more vulnerable to mortality and morbidity. It is also recommended that families receive breastfeeding support, counseling on danger signs, and exams of the infant and cord before discharge. Communication, consent, and respect for autonomy are important components of a positive experience of care. But, efforts to improve the quality of newborn care have often focused on provision of care with low or no priority on experience of care.[24] Research on experiences of newborn care (parental consent for procedures or exams, mistreatment and neglect, and separating infants from mothers or caregivers when not medically necessary) represents a notable gap in literature.

Evidence indicates that despite newborns' inability to exercise autonomy or use words to express their needs, positive experiences of care center around their relationship and connection with their mothers and caregivers. A strong mother-infant relationship forms the foundation for infant

For peer review only - http://bmjopen.bmj.com/site/about/guidelines.xhtml

health and development, and the period after birth is especially important.[25] Immediately following birth, infants display highly specialized attachment and bonding behaviour towards their mothers,[26,27] and sensory connections with mothers form a strong basis for the regulation of their physiological systems.[28] For example, research on skin-to-skin contact (SSC) after birth has shown that early sensory connections improve many physical, psychological, and care outcomes, such as maintenance of newborn temperature,[29] stable respiratory and cardiac function,[30] organization of sleep cycles,[31] development of neurophysiological systems,[32,33] and reducing newborn stress.[29,34] In contrast, separation of mothers and infants hinders attachment, induces stress and behaviors that alter physiological processes resulting in an altered developmental trajectory.[28,35]

This highlights newborns' needs to effectively bond with family, receive attentive care, and be kept warm and treated gently. Sensory cues from infants–suckling, sight, smell, sounds–also stimulate maternal neurobiological processes which subsequently affect maternal mental health and caregiving. [36–38] Evidence also suggests that the benefits of early bonding likely extends to other caregivers as well, such as fathers, with implications for future involvement in care.[39]

Fostering the mother-infant bond after birth also has profound implications for early and long-term breastfeeding. Mother-infant interactions stimulate maternal milk production and prepare infants to breastfeed by downregulating the neuroendocrine stress response and activating blood glucose regulation.[40,41] The evidence of a link between close maternal-infant contact and breastfeeding is compelling: mother-infant pairs who engaged in SSC after birth were more likely to adequately suckle during their first feed, continue breastfeeding after 1-4 months post-

For peer review only

birth, score higher on suckling competence, and breastfeed exclusively and for longer durations when compared to those without SSC.[29,42] This underscores the need to foster mother-infant interactions and minimize unnecessary interventions in maternity care.

Lack of information and informed consent have been cited as factors contributing to traumatic experiences and dissatisfaction in maternity care.[43–45] Lack of consent for women's care is regarded as an essential component of respectful care, but consent for procedures performed on newborns has rarely been studied. Because infants are completely dependent upon their caregivers, they are essential advocates to ensure safe and gentle medical care. Parents (or designated legal guardians) should be the decision-makers regarding their infants' care, and it is likely that women and families value the ability to make informed decisions and advocate for their newborns' care.

There are indications that poor treatment of newborns in facility-based maternity care occurs, including preliminary documentation of prevalence of certain types of occurrences. Recent literature has brought attention to the prevalence of practices such as unnecessary separation of newborns from mothers, neglect (absence of care), and non-consent for procedures.[7,10,46] However, these experiences have seldom been examined as primary indicators and available information has emerged from secondary data from other studies. A multi-country African study on treatment of women included observations of newborns in the first two hours after birth and found that over half of newborns were separated from their mothers within the first two hours, with higher prevalence among single and less educated women.[47] However, while some studies have documented high prevalence of these experiences and inequities in care, it remains

For peer review only

For peer review only - http://bmjopen.bmj.com/site/about/guidelines.xhtml

unclear how they are associated with other measures of quality care or outcomes (e.g. positive experiences of care, health behaviours, or health outcomes). Moreover, evidence suggests that person-centered maternity care is consequential for newborn health. One recent study in Kenya found that women's poor experiences of care were associated with increased report of newborn complications and lower likelihood of attending future medical visits.[48] To our knowledge, no previous studies have linked disrespectful neonatal care experiences, namely separation of newborns from mothers and lack of parental consent for newborn procedures, to health outcomes. This study aims to address these gaps.

Further research is needed to explore how newborn care experiences, and the implementation of institutional policies and provider practices, impact maternal and neonatal outcomes.[40,49] The aim of this study was to investigate the prevalence, risk factors, and outcomes associated with newborn care experiences, namely separating newborns from mothers and parental consent for newborn care, in several facilities in Kenya. In this study, we examined these practices using data from the Strengthening Person-Centered, Accessibility, Respectful Care, and Quality Study (SPARQ) in Kenya. In addition to investigating the extent to which these experiences occur, we sought to understand underlying risk factors and whether certain groups were at greater risk (i.e. 'discriminatory' practices). We also examined details surrounding newborn separation, including the duration and reasons for separation, and other newborn care practices such as SSC and breastfeeding support. Finally, we sought to understand the consequences of these experiences on maternal and newborn health outcomes: satisfaction with care, postpartum care attendance, and breastfeeding.

For peer review only - http://bmjopen.bmj.com/site/about/guidelines.xhtml

METHODS

Setting

This longitudinal study was conducted between September 2019 and January 2020 across six health facilities within Nairobi and Kiambu Counties, Kenya. The facilities varied in type (three public hospitals, two private hospitals, and one health centre/dispensary) and size (medium to large referral hospitals ranging from 100 to 900 reported deliveries per month). The facilities were conveniently selected based on their location within the two counties, reported deliveries of at least 100 per month, and the facility administration's willingness to participate in the study.

Data collection and recruitment

A team of twenty-four enumerators were involved in data collection, who had previous quantitative and research ethics training. The baseline survey was pre-tested with women who had recently delivered in four facilities (two of which were study facilities) to identify any issues with question flow or understanding via interviewing. The follow-up phone surveys were also pre-tested with this sample.

All surveys were administered in a private space within the facility to ensure confidentiality and privacy. Follow-up surveys were conducted by phone, ensuring privacy to maintain confidentiality.

Women were enrolled in the study postpartum when recovering in the maternity ward or at discharge before exiting the facility, all within 48 hours after birth. Women provided written

For peer review only

informed consent. Participants were also administered follow-up phone surveys between 2-4 weeks and/or 10 weeks postpartum. Participants were given airtime credit of approximately $2.00 for the baseline and $0.50 for each follow-up as a token of appreciation.

Analysis

The analytic sample included 1,014 women who completed baseline and at least one follow-up interview (Supplement 1). Data for exclusive breastfeeding were missing from 3 women in the 2-4 week follow-up and 4 women in the 10 week follow-up resulting in an analytic sample for exclusive breastfeeding of 829 and 839 participants in the 2-4 week and 10 week follow-up, respectively.

Variables examined are presented in Table 1. Our outcomes of interest included *Satisfaction with newborn care* measured at baseline, and *Attendance of post-partum check-up,* and *Exclusive breastfeeding* measured at follow-up. Primary independent variables were *Newborn separation, Newborn separation >10 minutes,* and *Consent for newborn procedures*. Although we did not find evidence in the literature about a critical duration of separation, we used a 10 minute threshold for separation as a proxy for short and long separation. Aside from *Perceived necessary separation*, additional probing questions were not asked. We examined descriptive characteristics of all women in the sample. We explored sociodemographic characteristics of the participants including age, parity, marital status, education, employment, woman's birthplace, insurance status, and facility type. We examined maternal health indicators of self-rated health status, maternal complications at delivery, and report of maternal complications since discharge. Newborn health characteristics were also explored, including birthweight, gestational age, and

For peer review only - http://bmjopen.bmj.com/site/about/guidelines.xhtml

report of newborn complications at baseline and after discharge. Lastly, we examined other measures of care received using a *Clinical quality index* and a *Breastfeeding care index* based on WHO standards of care.[23]

Table 1. Definition of variables

**Indicator**	**Question/Measure**	**Values**
Outcome Indicators		
Satisfaction with newborn care	In general, how satisfied were you with the services and care your newborn received after delivery? (collected at baseline)	"Satisfied or very satisfied" vs. "Dissatisfied or very dissatisfied"
Attendance of post-partum check-up	Have you attended any postpartum visits since being discharged? [1] (collected at follow-up)	"Yes" vs. "No"
Exclusive breastfeeding	How were you feeding your baby? [1] (collected at follow-up)	Fed infant with breastmilk only vs. fed with any other substances
Newborn separation and consent indicators (collected at baseline)		
Newborn separation	Was your baby ever separated from you by doctors, nurses, or other health providers for any reason?	"Yes" vs. "No"
Newborn separation >10 minutes	Was your baby ever separated from you for more than 10 minutes?	"Yes" vs. "No"
Perceived necessary separation	Did you ever feel that it was not necessary for your baby to be separated from you?	"Yes" vs. "No"
Consent sought for newborn procedures	Did the doctors, nurses, or other providers ask you for your permission before doing procedures or examinations on your baby?	"Yes" vs. "No"
Other variables (collected at baseline)		
Clinical quality index	Summative score comprising 7 newborn care questions indicating whether specific procedures were performed (skin-to-skin contact after birth; infant exam after delivery; infant dried after birth; delayed bathing; cord examination; temperature assessment; and mother/family counseled on newborn danger signs)	Range 0-7
Breastfeeding care index	Summative score of 3 indicators of breastfeeding support at the facility. (i.e. provider checked breastfeeding within two hours of delivery; mother/family counseled about breastfeeding; brestfeeding observed or shown.	Range 0-3

[1]Postpartum visit could have been for either maternal or newborn health.

For peer review only - http://bmjopen.bmj.com/site/about/guidelines.xhtml

Bivariate analyses investigated relationships between newborn care indicators and outcomes using chi-square and t-tests. We conducted multivariate logistic regression analyses assessing associations between newborn separation and consent indicators and described outcomes. Analyses adjusted for factors that may theoretically confound associations including age, parity, marital status, education, employment, birthplace, health facility type, insurance status, self-rated health, and clinical care index score. Analyses for *Satisfaction with newborn care* also adjusted for maternal and newborn complications at baseline and analyses for outcomes measured at follow-up interview were adjusted for maternal and newborn complications since discharge. Models for breastfeeding outcomes also adjusted for breastfeeding care index score. Cluster robust standard errors were used to account for clustering by facility. Model specification was tested using link tests and model fit was tested by Hosmer-Lemeshow goodness-of-fit tests. Sensitivity analyses examined potential effects of preterm birth and low birthweight, but no evidence of confounding was found. Analyses were performed using STATA/SE 15.1.

Ethics Approval

All study procedures were approved by the Institutional Review Boards at the University of California, San Francisco (protocol number 19-27783) and the Kenya Medical Research Institute (Protocol KEMRI Non-SSC 666). Informed consent was obtained from all participants prior to participation.

Patient and public involvement

Patients and the general community were not involved in the design, recruitment, or conduct of this study. During the consent process, participants were informed that they would not directly

For peer review only - http://bmjopen.bmj.com/site/about/guidelines.xhtml

benefit from their participation or be involved in the dissemination of the study results. Study results will be shared with facilities in order to improve their service delivery.

RESULTS

Descriptive characteristics of the sample of 1,014 women, stratified by reports of *newborn separation, newborn separation >10 minutes, and consent sought for newborn procedures,* are presented in Table 2.

Women who were separated from their newborn more than 10 minutes tended to be slightly older (p=0.036) and not born in Nairobi or Kiambu counties (p=0.043) compared to women reporting separation for less than 10 minutes or no separation. A greater proportion of women at government hospitals were separated from their newborns compared to women at government health clinics or private facilities (p<0.001). Delivery complications were also positively associated with newborn separation (p=0.004) and separation for more than 10 minutes (p=0.025).

Women who were asked permission for newborn procedures and examinations were older (p=0.026) and more likely to be covered under a health scheme or health insurance (p=0.028) than those who were not asked for permission.

Table 2. Descriptive characteristics of women included in the sample by reports of newborn separation, newborn separation for more than 10 minutes, and consent sought for newborn procedures (n=1,014)

For peer review only - http://bmjopen.bmj.com/site/about/guidelines.xhtml

Characteristics	Total	Newborn separated: Not separated	Newborn separated: Separated	Newborn separated: p-value	Newborn separated >10min: Not separated/ separated ≤10min	Newborn separated >10min: Separated >10min	Newborn separated >10min: p-value	Consent for newborn procedures[1]: Did not ask for permission	Consent for newborn procedures[1]: Asked for permission	Consent for newborn procedures[1]: p-value
Total number in group	1014	836	178		886	128		607	406	
Age (mean)	25.7	25.58	26.11	0.202	25.55	26.54	0.036	25.38	26.1	0.026
SD	(5.01)	(4.97)	(5.19)		(4.95)	(5.32)		(4.96)	(5.05)	
Parity (mean)	2.0	2.00	2.16	0.050	2.01	2.22	0.021	2.02	2.04	0.714
SD	(0.98)	(0.97)	(1.02)		(0.97)	(1.03)		(0.98)	(0.98)	
Multiparous										
No	35.7%	36.7%	30.9%	0.141	36.7%	28.9%	0.086	36.1%	35.2%	0.780
Yes	64.3%	63.3%	69.1%		63.3%	71.1%		63.9%	64.8%	
Currently married or partnered										
No	81.8%	18.7%	16.3%	0.458	18.2%	18.8%	0.874	19.1%	17.0%	0.393
Yes	18.2%	81.3%	83.7%		81.8%	81.2%		80.9%	83.0%	
Educational attainment										
Primary or less	43.7%	43.8%	43.3%	0.894	44.0%	41.4%	0.855	44.8%	41.9%	0.267
Vocational/	39.6%	39.4%	41.0%		39.4%	41.4%		40.0%	39.2%	
College/University	16.7%	16.9%	15.7%		16.6%	17.2%		15.2%	19.0%	
Currently employed										
No	59.5%	59.0%	61.8%	0.486	59.7%	57.8%	0.683	61.6%	56.4%	0.098
Yes	40.5%	41.0%	38.2%		40.3%	42.2%		38.4%	43.6%	
Born in Nairobi or Kiambu Counties										
No	78.0%	76.8%	83.7%	0.043	77.3%	82.8%	0.160	77.6%	78.6%	0.713
Yes	22.0%	23.2%	16.3%		22.7%	17.2%		22.4%	21.4%	
Self-rated health status										
Excellent or very good	34.7%	33.5%	40.4%	0.043	34.1%	39.1%	0.144	33.3%	36.9%	0.502
Good	40.4%	42.5%	30.9%		41.6%	32.0%		40.4%	40.4%	
Fair	15.7%	15.2%	18.0%		15.0%	20.3%		16.8%	14.0%	
Poor or very poor	9.2%	8.9%	10.7%		9.3%	8.6%		9.6%	8.6%	
Maternal Complications during delivery										
No	93.6%	94.6%	88.8%	0.004	94.2%	89.1%	0.025	92.9%	94.6%	0.289
Yes	6.4%	5.4%	11.2%		5.8%	10.9%		7.1%	5.4%	
Reported maternal complications at follow-up										
No	86.1%	86.4%	84.8%	0.592	86.0%	86.7%	0.827	85.3%	87.2%	0.403
Yes	13.9%	13.6%	15.2%		14.0%	13.3%		14.7%	12.8%	
Covered under health scheme or health insurance										
No	14.0%	13.9%	14.6%	0.799	13.9%	14.8%	0.77	16.0%	11.1%	0.028
Yes	86.0%	86.1%	85.4%		86.1%	85.2%		84.0%	88.9%	
Facility type										
Gov't hospital	73.9%	70.8%	88.2%	<0.001	71.7%	89.1%	<0.001	74.3%	73.2%	0.881
Gov't Health Centre/Dispensary	12.0%	13.9%	3.4%		13.4%	2.3%		12.0%	12.1%	
Private facility	14.1%	15.3%	8.4%		14.9%	8.6%		13.7%	14.8%	

[1] N=1,013 (1 refused to answer)

For peer review only

Table 3 displays newborn characteristics and other clinical and breastfeeding care indicators by reported experience of newborn separation and consent sought for newborn care. 17.6% of women reported being separated from their newborns while at the health facility. Among those women (n=178), the majority reported separation longer than 10 minutes (71.9%) and nearly half felt separation was unnecessary (44.9%). Most of those separated reported that their newborn was separated for procedures or examination (82.6%), but 8.4% reported that they did not know or were not told.[1] During separation, only one-in-five (19.6%) were able to have another parent, family member, or caregiver remain present with the newborn. Almost two-thirds of women (59.9%) reported that health providers did not ask permission before performing procedures or examinations on their newborn.

Mothers of newborns with complications were more likely to report separation than those who reported no complications ($p<0.001$). Mothers who were separated from their newborns were more likely to have their infant examined after delivery than not ($p=0.049$) but were less likely to have a provider: ask consent before newborn procedures or exams ($p=0.023$), check if breastfeeding was going well ($p=0.048$), or observe or demonstrate how to breastfeed ($p=0.013$). Almost all indicators of better clinical and breastfeeding care, such as skin-to-skin contact, newborn exam after delivery, newborn wiped, cord examination, temperature assessment, counseling on danger signs for newborns, and breastfeeding checks, counseling, and observation, were positively associated with consent sought for newborn procedures (Supplement 2). Among the full sample, the mean clinical quality index score was 4.14 (SD 1.37) out of 7. Those who reported being asked permission for newborn procedures had a mean clinical quality of care

[1] Of those who reported "Don't know," 80.0% reported being satisfied with newborn care. In comparison, 94.1% of the total sample reported being satisfied with newborn care.

For peer review only - http://bmjopen.bmj.com/site/about/guidelines.xhtml

index of 4.64 (SD 1.17) compared to 3.81(SD 1.38) among those who did not report being asked permission (p<0.001). The mean breastfeeding care index score among those who reported being asked permission was 2.3 (SD 0.94) out of 3 compared to 1.79 (SD 1.10) for those who reported not being asked permission (p<0.001).

Table 3. Distributions of newborn characteristics and newborn care indicators by reports of newborn separation and newborn consent indicators (n=1,014)

		Newborn separation			Newborn separation >10min			Consent sought for newborn procedures[1]		
**Variable**	**Total**	**Not separated**	**Separated**	**p-value**	**Not separated or separated ≤10min**	**Separated >10min**	**p-value**	**Did not ask for permission**	**Asked for permission**	**p-value**
Total number in group	1014	836	178		886	128		607	406	
**Health indicators**										
Birth weight										
Low BW (<2.5kg)	2.3%	2.2%	2.8%	0.002	2.3%	2.3%	<0.001	2.8%	1.5%	0.588
Normal BW (2.5-4.0kg)	89.3%	90.8%	82.6%		90.6%	80.5%		88.8%	90.2%	
Macrosomia (>4.0kg)	2.2%	2.2%	2.3%		2.1%	2.3%		1.7%	3.0%	
Missing	6.2%	4.9%	12.4%		5.0%	14.8%		6.8%	5.4%	
Preterm										
Not preterm (GA 38 weeks or more)	77.4%	77.2%	78.7%	0.418	77.3%	78.1%	0.266	77.9%	76.9%	<0.001
Yes, preterm (GA 37 weeks or less)	22.4%	22.7%	20.8%		22.6%	21.1%		21.9%	23.2%	
Missing	0.2%	0.1%	0.6%		0.1%	0.8%		0.2%	0.0%	
Newborn complications reported at baseline										
Healthy, no issues	96.3%	97.5%	90.5%	<0.001	97.5%	87.5%	<0.001	96.4%	96.1%	0.929
Complications	3.6%	2.4%	9.6%		2.4%	12.5%		3.5%	3.9%	
Missing[2]	0.1%	0.1%	0.0%		0.1%	0.0%		0.2%	0.0%	
Newborn complications reported since discharge										
No complications	67.8%	68.2%	65.7%	0.525	68.1%	65.6%	0.582	68.0%	67.2%	0.790
Complications	32.3%	31.8%	34.3%		31.9%	34.4%		32.0%	32.8%	
**Newborn care indicators**										
Newborn separation										
No	82.4%	---	---		---	---		80.9%	85.%	0.093
Yes	17.6%	---	---		---	---		19.1%	15.%	
Consent sought for newborn procedures										
No	59.9%	58.7%	65.2%	0.023	58.9%	66.4%	0.007	---	---	
Yes	40.0%	41.3%	34.3%		41.1%	32.8%		---	---	
Refused to answer	0.1%	0.0%	0.6%		0.0%	0.8%		---	---	
**Of those who were separated from newborns (n=178)**										
Newborn separation > 10 min										
No	4.9%	---	28.1%		---	---		26.7%	31.1%	0.534
Yes	12.6%	---	71.9%		---	---		73.3%	68.9%	

Was your baby separated from you by doctors, nurses, or other health providers to perform procedures and examinations?

For peer review only

Variable	Total	Newborn separation: Not separated	Separated	p-value	Newborn separation >10min: Not separated or separated ≤10min	Separated >10min	p-value	Consent sought for newborn procedures[1]: Did not ask for permission	Asked for permission	p-value
No	1.6%	---	9.0%		14.0%	7.0%	0.344	8.6%	9.8%	0.868
Yes	14.5%		82.6%		78.0%	84.4%		82.8%	83.6%	
Don't know	1.5%		8.4%		8.0%	8.6%		8.6%	6.6%	
Did you ever feel that it was not necessary for your baby to be separated from you?										
No	9.7%	---	55.1%		60.0%	53.1%	0.407	53.4%	59.0%	0.479
Yes	7.9%	---	44.9%		40.0%	46.9%		46.6%	41.0%	
If you were ever unable to be with your newborn, was another parent, family members, or caregiver able to be with your baby?										
No	14.2%	---	80.9%		78.0%	82.%	0.539	87.1%	68.9%	0.003
Yes	12.6%	---	19.1%		22.0%	18.%		12.9%	31.1%	
**Other care indicators**										
Clinical quality index										
Mean	4.14	4.14	4.15	0.965	4.13	4.20	0.637	3.81	4.64	<0.001
SD	(1.37)	(1.35)	(1.45)		(1.36)	(1.40)		(1.38)	(1.17)	
Breastfeeding care index										
Mean	2.00	2.04	1.80	0.006	2.01	1.88	0.173	1.79	2.30	<0.001
SD	(1.07)	(1.05)	(1.13)		(1.06)	(1.10)		(1.10)	(0.94)	

[1] Sample size, N=1,013 (1 refused to answer)
[2] For analyses, one missing response for newborn complications was recoded as "healthy, no issues."

The distributions of outcomes by reports of newborn separation, separation for more than 10 minutes, and consent sought for newborn procedures are presented in Table 4. Women who were asked permission for newborn procedures were more likely to be satisfied with care (p<0.001) and attend a postpartum visit after discharge (p<0.001) than those who were not asked permission.

Table 4. Distributions of outcomes by reports of newborn separation, separation for more than 10 minutes, and consent sought for newborn procedures (n=1,014)

Outcomes	Total	Newborn separated: Not separated	Separated	p-value	Newborn separated >10min: Not separated or separated ≤ 10min	Separated >10min	p-value	Consent sought for newborn procedures[1]: Did not ask for permission	Asked for permission	p-value
Total number in group	1014	836	178		886	128		607	406	
Satisfaction with newborn care										

For peer review only - http://bmjopen.bmj.com/site/about/guidelines.xhtml

Dissatisfied	5.9%	6.1%	5.1%	0.592	6.1%	4.7%	0.528	8.4%	2.0%	<0.001
Satisfied	94.1%	93.9%	94.9%		93.9%	95.3%		91.6%	98.0%	
Attended postpartum visit after discharge, within 10 weeks										
No, did not attend	53.1%	53.5%	51.1%	0.569	53.4%	50.8%	0.581	57.7%	46.1%	<0.001
Yes, attended	46.9%	46.5%	48.9%		46.6%	49.2%		42.3%	53.9%	
Exclusively breastfeeding at 2-4 week follow-up [2]										
No	9.0%	8.5%	11.9%	0.193	8.5%	13.%	0.128	9.8%	8.0%	0.373
Yes	91.0%	91.5%	88.1%		91.5%	87.%		90.2%	92.0%	
Exclusively breastfeeding at 10 week follow-up [3]										
No	18.0%	17.7%	19.6%	0.577	17.6%	21.0%	0.399	20.1%	15.0%	0.058
Yes	82.0%	82.3%	80.4%		82.4%	79.0%		79.9%	85.0%	

[1] Sample size, N=1,013 (1 refused to answer)
[2] Sample size, N=829 (women who completed 2-4 week follow-up and reported on exclusive breastfeeding)
[3] Sample size, N=839 (women who completed 10 week follow-up and reported on exclusive breastfeeding)

Results of multivariate analyses examining associations between reports of newborn separation, consent sought for newborn procedures, and health outcomes are presented in Table 5 (full models are presented in Supplement 3). Among women reporting any newborn separation during care, there was a negative to no association with exclusive breastfeeding at 2-4 weeks (aOR=0.67, 95%CI: 0.41, 1.09). However, women who reported separation from their newborn longer than 10 minutes were 44% less likely to be exclusively breastfeeding at 2-4 weeks compared to those reporting less than 10 minutes or no separation (aOR=0.56, 95%CI: 0.40, 0.76). No associations were found with satisfaction with newborn care, postpartum visit attendance, or exclusive breastfeeding at 10 weeks.

Reported consent sought for newborn procedures was positively associated with satisfaction with newborn care at baseline, attending a postpartum visit by 10 weeks, and exclusive breastfeeding at 10 weeks. Women who reported providers asked for permission before performing newborns' procedures or examinations were 2.7 times more likely to be satisfied with care (aOR=2.71,

For peer review only - http://bmjopen.bmj.com/site/about/guidelines.xhtml

95%CI: 1.67, 4.41) and were also 27% more likely to attend a postpartum visit by either 2-4 weeks or 10 weeks (aOR=1.27, 95%CI: 1.05, 4.41) after controlling for covariates. Report of consent for newborn procedures displayed no association with exclusive breastfeeding at 2-4 weeks (aOR=1.06, 95%CI: 0.78, 1.44), but was associated with a 33% increased likelihood of exclusive breastfeeding at 10 weeks (aOR=1.33, 95%CI: 1.10, 1.62) even after controlling for breastfeeding care index, and other previously described covariates.

Table 5. Results of multivariate logistic regression analyses examining the associations between separation, consent for newborn care, and maternal and newborn outcomes (n=1,014)

	**Newborn separated from mother**	**Newborn separated for more than 10 minutes**	**Consent sought for newborn procedures [1]**
**Outcome**	aOR (95% CI)	aOR (95% CI)	aOR (95% CI)
Satisfaction with newborn care at baseline *(ref. not satisfied)*	1.34 (0.75, 2.41)	1.36 (0.57, 3.28)	2.71 (1.67, 4.41)***
Attended a post-partum visit, within 10 weeks *(ref. no visit)*	1.14 (0.96, 1.35)	1.11 (0.86, 1.43)	1.27 (1.05, 1.55)*
Exclusively breastfeeding at 2-4 weeks [2] *(ref. not exclusively breastfed)*	0.67 (0.41, 1.09)	0.56 (0.40, 0.76)**	1.06 (0.78, 1.44) [3]
Exclusively breastfeeding at 10 weeks [4] *(ref. not exclusively breastfed)*	0.76 (0.49, 1.18)	0.66 (0.39, 1.10)	1.33 (1.10, 1.62)**

Note: All estimates of odds ratios (and 95% confidence intervals) were adjusted for age, multiparity, marital status, education, employment, women's birthplace, facility type, insurance status, health status, and clinical quality of care index. Satisfaction with newborn care models also adjusted for maternal complications and newborn complications at baseline. Breastfeeding outcome models also adjusted for breastfeeding care index, newborn complications at baseline, and both newborn and maternal complications after discharge. Post-partum visit models also adjusted for maternal and newborn complications after discharge. Cluster robust standard errors were used to account for clustering by facility.
[1] Sample size, N=1,013 (women who reported consent sought for newborn procedures, excluded 1 missing response)

For peer review only - http://bmjopen.bmj.com/site/about/guidelines.xhtml

[2] Sample size, N=829 (women who completed 2-4 week follow up survey and reported exclusive breastfeeding status)
[3] Sample size, N=828 (women who completed 2-4 week follow up survey and reported exclusive breastfeeding status, excluded 1 missing response for consent sought for newborn procedures)
[4] Sample size, N=839 (women who completed 10 week follow up survey and reported exclusive breastfeeding status)
*p<0.05, **p<0.01, ***p<0.001

DISCUSSION

The immediate postpartum period is a critical clinical period with high mortality risk, and an important social period for maternal-newborn bonding. Whether positive or negative, most families will hold strong memories of experiences in this time, and it may impact their opinions about and willingness to seek care in the future, health practices in the home, and long-term health outcomes. We sought to examine two newborn care experiences related to the experience of care, to understand the context of these experiences, and to assess associations with relevant outcomes: care satisfaction, use and breastfeeding. This study provides evidence that treatment of newborns is an important component of quality and is likely to influence newborn health and postpartum practices.

Importantly, this study found significant inequities related to treatment of women and their newborns. For example, being consented for newborn care was correlated with insurance coverage, indicating that poorer women and their newborns may not be treated equally by the health system with regard to their clinical care. Women with higher parity, born outside of Nairobi or Kiambu counties, and who experienced delivery complications were more likely to experience separation from their newborns. Mothers and newborns were most likely to be separated at government hospitals, where the most women in Kenya deliver. Although less common, separation did occur in the smaller health centre/dispensary as well as private facilities.

For peer review only - http://bmjopen.bmj.com/site/about/guidelines.xhtml

Other studies have found that while larger hospitals provide better clinical quality care than smaller hospitals, women are also more likely to experience poor patient-centered care.[50] Further research is needed to understand how much of separation is driven by facility policies rather than individual provider behaviour. Infrastructural issues like lack of space and insufficient infection control procedures may influence policies at health facilities to limit family member entry or separate newborns.[51] In underfunded facilities, fatigue and stress of health workers likely influences their ability to provide high-quality care.[52,53] On the other hand, providers may have been trained to promote practices that are no longer evidenced-based, such as misconceptions around separation for infection control.[54] Although drivers of poor quality and disrespectful care are not fully understood,[46] these findings suggest that they are likely multi-faceted and interactive. More research is needed in a wider range of settings and countries, to explore the potential contextual factors that might influence policies, behaviours, and solutions.

In this study, 17.6% of women reported being separated from their newborns, which is less than has been reported in other studies.[47] Even so, many of these occurrences were thought by women to be unnecessary, and almost 10% were not given a reason for separation. Most separations were over 10 minutes and in only one-fifth was another family member able to accompany the newborn. In many contexts, and Kenya, specifically, fathers have limited access to maternity and postnatal wards and neonatal intensive care units.[8] Notably, other studies have found that women in Nairobi were less likely to want a companion during labor and delivery compared to women in rural Kenya, Ghana, and India; and were also less likely to report being allowed a companion all the time.[8,12] However, there is a potentially important role for

For peer review only - http://bmjopen.bmj.com/site/about/guidelines.xhtml

fathers, family members, or doulas, in staying with the newborn when mothers require rest, surgery, or critical care.[27] Further research is needed to examine facility-level policies in allowing a companion of choice and facilitating family support when it is desired.

Alarmingly, over 60% of women reported not being consented for newborn procedures. Report of being asked to provide consent was correlated with higher scores on the clinical quality of care and breastfeeding indices. This may reflect that providers or facilities that are more consistent in the provision of high quality care are also more likely to seek permission for procedures. This is consistent with another study in Kenya that found high levels of failure to provide informed consent and notable patient-provider discordance in reports of informed consent. Even in instances where providers reported obtaining consent from women for medical procedures, women were not provided full information or did not comprehend the information given to them.[14] Future research may also examine the extent to which consent practices were due to provider characteristics, such as position, level of training, or age.

We found that for both satisfaction and postpartum care utilization, report of being asked for permission for newborn care procedures was positively associated, further illustrating the importance of consent as an aspect of respectful care.

For peer review only

This study provides evidence that newborn treatment during childbirth is associated with newborn health and postpartum practices. Reported separation of newborns from their mothers for over 10 minutes was associated with decreased breastfeeding at a later time point. While this association could be confounded by newborns with more severe complications–which require additional procedures, and also affect breastfeeding–the association remained after adjustment in

For peer review only - http://bmjopen.bmj.com/site/about/guidelines.xhtml

the model. Sensitivity analyses found no effect of low birthweight and preterm birth on estimates of association (and neither were statistically significant in models), suggesting that this association was not due to smaller or sicker infants who may have greater difficulty breastfeeding. We also acknowledge the possibility that newborns may have been separated after the interview if they experienced later complications. While this study did not capture this information, we expect that this might have biased the estimated association to be more conservative.

Although the timing and duration of separation is likely important, we lacked information about the length of separation and when it occurred. Nevertheless, this finding suggests that any prolonged separation during the immediate postpartum period (within 48hrs) may inhibit critical bonding and breastfeeding processes. We highlight the need to investigate a critical threshold of separation and timing as an important area for future research.

It is also plausible that the lack of early initiation of breastfeeding and lower satisfaction with immediate postnatal care contributed to poorer breastfeeding practices after discharge because of lower motivation or encouragement. In addition to improved satisfaction and likelihood to utilize postnatal care, report of consent for newborn procedures was associated with a greater likelihood of exclusive breastfeeding at follow-up, indicating a potential causal link. In addition, given that women who did not report consent also reported lower clinical quality and breastfeeding care scores, these women likely experienced overall lower quality of care. These combined negative experiences of care may have also contributed to distrust of health professionals' advice to exclusively breastfeed or greater biological stress responses that may impede breastfeeding. This finding is important given the overwhelming evidence of the benefits of breastmilk for newborn survival, development and health of infants.[55,56] This adds to the

For peer review only

For peer review only - http://bmjopen.bmj.com/site/about/guidelines.xhtml

evidence found by other studies which showed that lack of respectful and supportive care during intrapartum care is associated with more reports of newborn complications.[48]

Strengths and limitations

This study is one of the first studies to include questions with the primary aim of understanding details around newborn separation, and to correlate newborn care experiences with subsequent health outcomes. While previous studies have reported on prevalence of newborn separation, [7,10,46] this study is the first to differentiate separation by maternal perception of reason and need, highlighting both the probability that much of the separation is not medically necessary, and that many parents do not fully understand the reasons for separation most likely due to poor communication. This study also goes beyond measuring maternal consent for care of self to consent of procedures for the newborn. Lastly, this study used longitudinal data and was able to measure treatment of newborns from pre-discharge care in facilities to postpartum practices.

We lacked information on the details of separation that may influence the magnitude of associations, including the duration or timing of separation and information about what types of newborn procedures or examinations women reported lack of consent, such as whether they were routine, invasive, emergency procedures or involved admission to a newborn ward. This study was limited to 10 weeks of follow up, and it is unknown how care practices or experience of care for newborns may influence care throughout infancy. Additionally, the samples for the 2-4 and 10 week follow-ups differed slightly (though not significantly across socio-demographics or care), so our comparisons of exclusive breastfeeding between these time periods may be

For peer review only - http://bmjopen.bmj.com/site/about/guidelines.xhtml

interpreted conservatively due to smaller sample size and potential biases due to composition. Further, this study focused on women's reports of their care and did not triangulate with hospital records or observations. Thus, participants may have had recall bias or notable reporting error and results should be interpreted conservatively.[57] The negative framing of newborn experience questions may have also biased participants to respond more negatively to other questions, such as satisfaction. Participants may have defined "separation" differently than the researchers, and may have different interpretations than health care providers about certain practices, such as necessity of separation. While it was explained to participants that this study was confidential and was being conducted independently of the facility or health system, there may have been desirability bias if respondents were concerned about their subsequent care at facilities. Lastly, our results may have limited generalizability, as our study sample included facilities from Nairobi and Kiambu Counties, Kenya. Future research may explore whether associations with these care experiences are also present in other social and institutional contexts.

Conclusions

Newborns, their mothers, and their families have a right to high quality, respectful care, regardless of their characteristics or where they are delivered. High quality newborn care includes the ability to stay together and for the parents to be informed and consent for care. The implications of various practices on health outcomes a month or more after discharge illustrate the importance of a positive experience of postnatal care. More research and action are needed to provide newborns with the standard of care that they deserve.

Contributors

For peer review only

For peer review only - http://bmjopen.bmj.com/site/about/guidelines.xhtml

MS and ES conceptualized and designed the study. MN analyzed the data. MN, ES, JO, and MS contributed to the writing and revision of the manuscript.

Acknowledgements

We would like to thank Innovations for Poverty Action – Kenya for their contributions to the conceptualization and data collection. We thank Ginger Golub and Sun Cotter for assistance in collection of data. We also express gratitude and appreciation to all of the women who participated in this study.

Data Sharing

Data are available upon reasonable request. Please contact the corresponding author with requests for de-identified participant data.

Funding statement

This study was funded by the Bill and Melinda Gates Foundation (OPP1127467). The authors also thank the California Center for Population Research at UCLA (CCPR) for its training support from the Eunice Kennedy Shriver National Institute of Child Health and Human Development (T32-HD007545).

Competing interests

None declared.

For peer review only

References

1. UN IGME. Levels & Trends in Child Mortality: Report 2020, Estimates developed by the UN Inter-agency Group for Child Mortality Estimation [Internet]. New York; 2020.
2. HNN. Kenya Data [Internet]. Healthy Newborn Network. 2020 [cited 2020 Aug 6]. Available from: https://www.healthynewbornnetwork.org/country/kenya/
3. UN IGME. Kenya Neonatal Mortality Estimates [Internet]. UN IGME. 2018 [cited 2020 Aug 6]. Available from: https://childmortality.org/data/Kenya
4. WHO, UNICEF. Every Newborn: an action plan to end preventable deaths [Internet]. Geneva; 2014.
5. Tunçalp, Were WM, Maclennan C, Oladapo OT, Gülmezoglu AM, Bahl R, et al. Quality of care for pregnant women and newborns - The WHO vision. BJOG An Int J Obstet Gynaecol. 2015 Jul 1;122(8):1045–9.
6. Rosen HE, Lynam PF, Carr C, Reis V, Ricca J, Bazant ES, et al. Direct observation of respectful maternity care in five countries: a cross-sectional study of health facilities in East and Southern Africa. BMC Pregnancy Childbirth [Internet]. 2015 Dec 23;15(1):306.
7. Reis V, Deller B, Carr C, Smith J. Respectful maternity care. Country experience [Internet]. Alliance francophone pour l'accouchement respecté (AFAR); 2016.
8. Afulani P, Phillips B, Aborigo RA, Moyer CA. Person-centred maternity care in low-income and middle-income countries: analysis of data from Kenya, Ghana, and India. Lancet Glob Heal. 2019 Jan 1;7(1):e96–109.
9. McMahon SA, George AS, Chebet JJ, Mosha IH, Mpembeni RN, Winch PJ. Experiences of and responses to disrespectful maternity care and abuse during childbirth; a qualitative study with women and men in Morogoro Region, Tanzania. BMC Pregnancy Childbirth [Internet]. 2014 Dec 12;14(1):268.
10. Bohren MA, Mehrtash H, Fawole B, Maung TM, Balde MD, Maya E, et al. How women are treated during facility-based childbirth in four countries: a cross-sectional study with labour observations and community-based surveys. Lancet. 2019 Nov 9;394(10210):1750–63.
11. Bohren MA, Hunter EC, Munthe-Kaas HM, Souza JP, Vogel JP, Gülmezoglu AM. Facilitators and barriers to facility-based delivery in low- and middle-income countries: A qualitative evidence synthesis. Vol. 11, Reproductive Health. BioMed Central Ltd.; 2014.
12. Afulani P, Kusi C, Kirumbi L, Walker D. Companionship during facility-based childbirth: results from a mixed-methods study with recently delivered women and providers in Kenya. BMC Pregnancy Childbirth [Internet]. 2018 Dec 10;18(1):150.
13. Kabakian-Khasholian T, Portela A. Companion of choice at birth: Factors affecting implementation. BMC Pregnancy Childbirth [Internet]. 2017 Aug 31;17(1):265.
14. Sudhinaraset M, Giessler K, Golub G, Afulani P. Providers and women's perspectives on person-centered maternity care: A mixed methods study in Kenya [Internet]. Vol. 18, International Journal for Equity in Health. BioMed Central Ltd.; 2019. p. 83.
15. Afulani P, Kirumbi L, Lyndon A. What makes or mars the facility-based childbirth experience: Thematic analysis of women's childbirth experiences in western Kenya. Reprod Health [Internet]. 2017 Dec 29;14(1):180.
16. Downe S, Finlayson K, Oladapo O, Bonet M, Gülmezoglu AM. What matters to women during childbirth: A systematic qualitative review [Internet]. Vol. 13, PLoS ONE. Public Library of Science; 2018. p. e0194906.
17. Brüggemann OM, Osis MJD, Parpinelli MA. Support during childbirth: Perception of

For peer review only

health care providers and companions chosen by women. Rev Saude Publica. 2007;41(1):1–9.
18. Owiti A, Oyugi J, Essink D. Utilization of Kenya's free maternal health services among women living in Kibera slums: A cross-sectional study. Pan Afr Med J. 2018;30.
19. Escamilla V, Calhoun L, Winston J, Speizer IS. The Role of Distance and Quality on Facility Selection for Maternal and Child Health Services in Urban Kenya. J Urban Heal. 2018 Feb 1;95(1).
20. Keats EC, Ngugi A, Macharia W, Akseer N, Khaemba EN, Bhatti Z, et al. Progress and priorities for reproductive, maternal, newborn, and child health in Kenya: a Countdown to 2015 country case study. Lancet Glob Heal. 2017 Aug 1;5(8):e782–95.
21. Sharma J, Leslie HH, Kundu F, Kruk ME. Poor quality for poor women? Inequities in the quality of antenatal and delivery care in Kenya. PLoS One. 2017 Jan 1;12(1).
22. Kruk ME, Pate M. The Lancet Global Health Commission on High Quality Health Systems 1 year on: progress on a global imperative [Internet]. Vol. 8, The Lancet Global Health. Elsevier Ltd; 2020. p. e30–2.
23. WHO. WHO standards of care to improve maternal and newborn quality of care in facilities [Internet]. Geneva; 2016.
24. Sacks E, Kinney M V. Respectful maternal and newborn care: Building a common agenda [Internet]. Vol. 12, Reproductive Health. BioMed Central Ltd.; 2015.
25. Klaus MH. Commentary: An Early, Short, and Useful Sensitive Period in the Human Infant. Birth [Internet]. 2009 Jun 1;36(2):110–2.
26. Sullivan R, Perry R, Sloan A, Kleinhaus K, Burtchen N. Infant Bonding and Attachment to the Caregiver: Insights from Basic and Clinical Science [Internet]. Vol. 38, Clinics in Perinatology. NIH Public Access; 2011. p. 643–55.
27. Widström A, Brimdyr K, Svensson K, Cadwell K, Nissen E. Skin-to-skin contact the first hour after birth, underlying implications and clinical practice. Acta Paediatr [Internet]. 2019 Jul 13;108(7):1192–204.
28. Bergman N. Birth practices: Maternal-neonate separation as a source of toxic stress [Internet]. Vol. 111, Birth Defects Research. John Wiley and Sons Inc.; 2019. p. 1087–109.
29. Stevens J, Schmied V, Burns E, Dahlen H. Immediate or early skin-to-skin contact after a Caesarean section: A review of the literature [Internet]. Vol. 10, Maternal and Child Nutrition. Blackwell Publishing Ltd; 2014. p. 456–73.
30. Ludington-Hoe SM, Swinth JY. Developmental aspects of kangaroo care. J Obstet Gynecol Neonatal Nurs [Internet]. 1996;25(8):691–703.
31. Feldman R, Eidelman AI. Skin-to-skin contact (Kangaroo Care) accelerates autonomic and neurobehavioural maturation in preterm infants. Dev Med Child Neurol. 2003 Apr 1;45(4):274–81.
32. Loman MM, Bruce J, Dallman M, Dozier M, Fisher P, Fox N, et al. Early Experience and the Development of Stress Reactivity and Regulation in Children Gunnar [on behalf of the Early Experience, Stress, and Neurobehavioral Development Center. Neurosci Biobehav Rev. 2010;34(6):867–76.
33. Champagne F, Meaney MJ. Like mother, like daughter: Evidence for non-genomic transmission of parental behavior and stress responsivity. In: Progress in Brain Research. Elsevier; 2001. p. 287–302.
34. Shonkoff JP, Garner AS, Siegel BS, Dobbins MI, Earls MF, McGuinn L, et al. The

For peer review only

For peer review only - http://bmjopen.bmj.com/site/about/guidelines.xhtml

lifelong effects of early childhood adversity and toxic stress. Pediatrics [Internet]. 2012 Jan;129(1).
35. Hofer MA. Psychobiological Roots of Early Attachment. Curr Dir Psychol Sci [Internet]. 2006 Apr 22;15(2):84–8.
36. Strathearn L, Mamun AA, Najman JM, O'Callaghan MJ. Does breastfeeding protect against substantiated child abuse and neglect? A 15-year cohort study. Pediatrics [Internet]. 2009 Feb;123(2):483–93.
37. Bigelow A, Power M, Maclellan-Peters J, Alex M, Mcdonald C. Effect of Mother/Infant Skin-to-Skin Contact on Postpartum Depressive Symptoms and Maternal Physiological Stress. JOGNN - J Obstet Gynecol Neonatal Nurs [Internet]. 2012;41(3):369–82.
38. Swain JE, Lorberbaum JP, Kose S, Strathearn L. Brain basis of early parent-infant interactions: Psychology, physiology, and in vivo functional neuroimaging studies. J Child Psychol Psychiatry Allied Discip [Internet]. 2007 Mar;48(3–4):262–87.
39. Scism AR, Cobb RL. Integrative Review of Factors and Interventions That Influence Early Father–Infant Bonding. Vol. 46, JOGNN - Journal of Obstetric, Gynecologic, and Neonatal Nursing. Elsevier B.V.; 2017. p. 163–70.
40. Hallowell SG, Froh EB, Spatz DL. Human milk and breastfeeding: An intervention to mitigate toxic stress. Nurs Outlook [Internet]. 2017 Jan 1;65(1):58–67.
41. Uvnäs-Moberg K. Neuroendocrinology of the mother-child interaction [Internet]. Vol. 7, Trends in Endocrinology and Metabolism. Elsevier Inc.; 1996. p. 126–31.
42. Moore ER, Bergman N, Anderson GC, Medley N. Early skin-to-skin contact for mothers and their healthy newborn infants [Internet]. Vol. 2016, Cochrane Database of Systematic Reviews. John Wiley and Sons Ltd; 2016.
43. Koster D, Romijn C, Sakko E, Stam C, Steenhuis N, de Vries D, et al. Traumatic childbirth experiences: practice-based implications for maternity care professionals from the woman's perspective. Scand J Caring Sci [Internet]. 2019;
44. Jolly Y, Aminu M, Mgawadere F, Van Den Broek N. "We are the ones who should make the decision" - Knowledge and understanding of the rights-based approach to maternity care among women and healthcare providers. BMC Pregnancy Childbirth [Internet]. 2019 Feb 15;19(1).
45. Shakibazadeh E, Namadian M, Bohren MA, Vogel JP, Rashidian A, Nogueira Pileggi V, et al. Respectful care during childbirth in health facilities globally: a qualitative evidence synthesis [Internet]. Vol. 125, BJOG: An International Journal of Obstetrics and Gynaecology. Blackwell Publishing Ltd; 2018. p. 932–42.
46. Sacks E. Defining disrespect and abuse of newborns: A review of the evidence and an expanded typology of respectful maternity care [Internet]. Vol. 14, Reproductive Health. BioMed Central Ltd.; 2017. p. 66.
47. Sacks E, Mehrtash H, Bohren MA, Balde MD, Vogel JP, Adu-Bonsaffoh K, et al. The first two hours after birth: prevalence and factors associated with newborn care practices from a multi-country, facility-based observational study. Lancet Glob Heal. 2020;in press.
48. Sudhinaraset M, Landrian A, Afulani P, Diamond-Smith N, Golub G. Association between person-centered maternity care and newborn complications in Kenya. Int J Gynecol Obstet [Internet]. 2020 Jan 9;148(1):27–34.
49. Strathearn L. Maternal neglect: Oxytocin, dopamine and the neurobiology of attachment [Internet]. Vol. 23, Journal of Neuroendocrinology. J Neuroendocrinol; 2011. p. 1054–65.
50. Phillips B, Kajal F, Montagu D, Kumar A, Kumar V. Is bigger better? Assessment of self-

For peer review only

reported and researcher-collected data on maternal health care quality among high-case-load facilities in Uttar Pradesh: a mixed-methods study. Lancet Glob Heal [Internet]. 2018 Mar 1;6:S46.

51. Sacks E, Sripad P, Ndwiga C, Waiswa P, Warren CE. Protecting newborn infants during the COVID-19 pandemic should be based on evidence and equity. Acta Paediatr [Internet]. 2020;

52. Mgawadere F, Smith H, Asfaw A, Lambert J, Broek N van den. “There is no time for knowing each other”: Quality of care during childbirth in a low resource setting. Midwifery [Internet]. 2019 Aug 1;75:33–40.

53. O’Donnell E, Utz B, Khonje D, van den Broek N. “At the right time, in the right way, with the right resources”: Perceptions of the quality of care provided during childbirth in Malawi. BMC Pregnancy Childbirth [Internet]. 2014 Jul 28;14(1):248.

54. Maimbolwa MC, Sikazwe N, Yamba B, Diwan V, Ransjö-Arvidson AB. Views on involving a social support person during labor in Zambian maternities. J Midwifery Womens Health [Internet]. 2001;46(4):226–34.

55. Lyons KE, Ryan CA, Dempsey EM, Ross RP, Stanton C. Breast Milk, a Source of Beneficial Microbes and Associated Benefits for Infant Health. Nutrients [Internet]. 2020 Apr 9;12(4):1039.

56. Paduraru D. The evidence for the benefits from breast milk in the neurodevelopment of premature babies – a review of the recent literature. J Mind Med Sci [Internet]. 2018 Oct 3;5(2):151–7.

57. Day LT, Sadeq-ur Rahman Q, Ehsanur Rahman A, Salim N, KC A, Ruysen H, et al. Assessment of the validity of the measurement of newborn and maternal health-care coverage in hospitals (EN-BIRTH): an observational study. Lancet Glob Heal. 2021 Mar 1;9(3):e267–79.

For peer review only

For peer review only - http://bmjopen.bmj.com/site/about/guidelines.xhtml

Supplement 1. Flowchart of baseline and follow-up analytic sample sizes

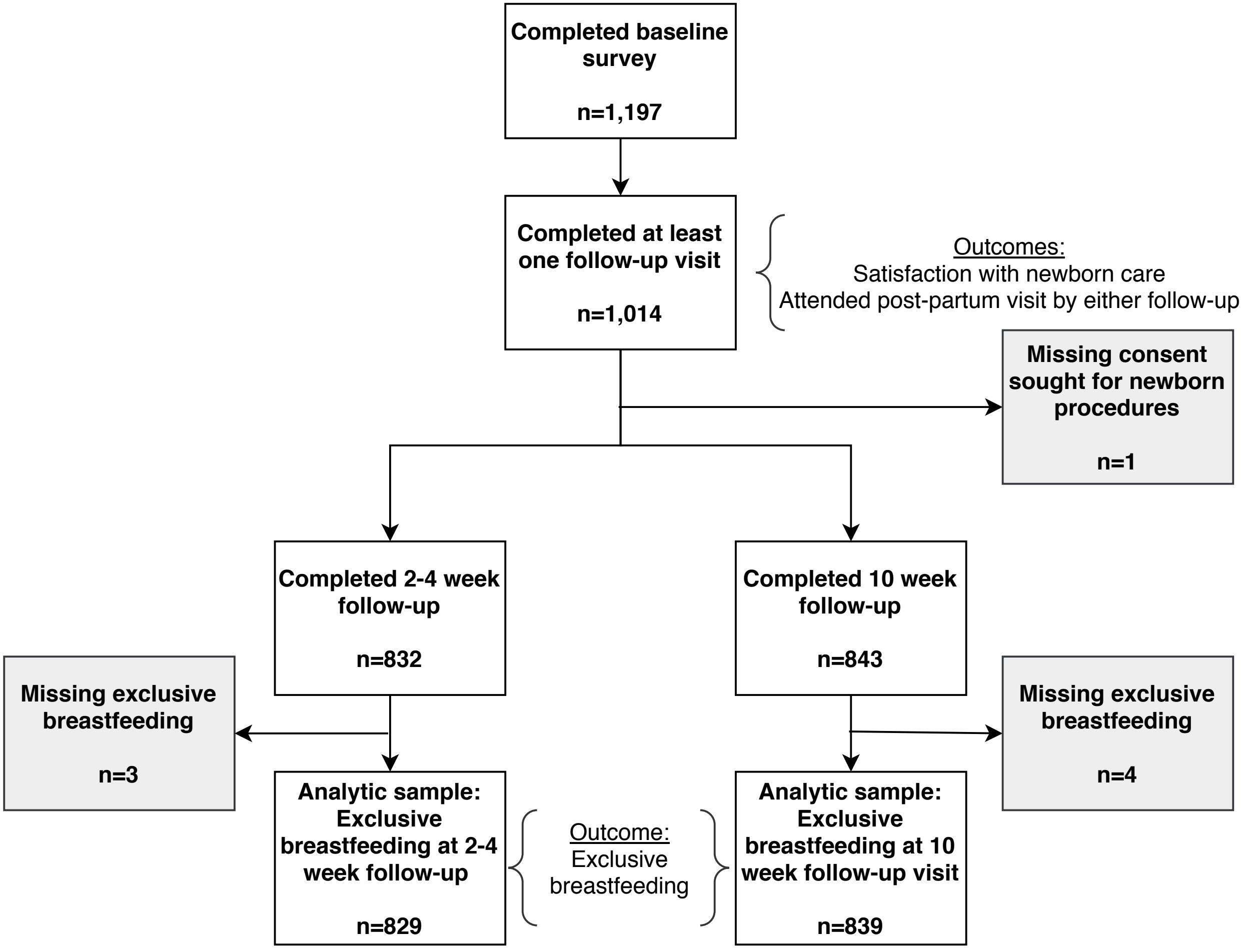

Supplement 2. Distributions of clinical and breastfeeding care indicators by reports of newborn separation and newborn consent indicators, (n=1,014)

		Newborn separation			Newborn separation >10min			Consent sought for newborn procedures[1]		
Variable	Total	Not separated	Separated	p-value	Not separated or separated ≤ 10min	Separated >10min	p-value	Did not ask for permission	Asked for permission	p-value
Total number in group	1,014	836	178		886	128		607	406	
**Clinical quality of care indicators**										
Was your baby put on your abdomen or chest as soon as it was born?										
No	13.6%	13.0%	16.3%	0.467	13.0%	18.0%	0.287	15.8%	10.3%	0.032
Yes	86.3%	86.8%	83.7%		86.9%	82.0%		84.0%	89.7%	
Don't know	0.1%	0.1%	0.0%		0.1%	0.0%		0.2%	0.0%	
Within two hours of delivery, did a health provider examine your baby?										
No	25.0%	25.7%	21.9%	0.049	26.4%	15.6%	0.001	31.3%	15.5%	<0.001
Yes	74.1%	73.7%	75.8%		73.0%	81.2%		67.5%	84.%	
Don't know	0.8%	0.6%	1.7%		0.6%	2.3%		1.2%	0.2%	
Missing	0.1%	0.0%	0.6%		0.0%	0.8%		0.0%	0.2%	
Was your baby wiped dry within a few minutes after birth?										
No	7.4%	7.3%	7.9%	0.013	7.2%	8.6%	0.091	9.2%	4.7%	0.003
Yes	90.4%	91.1%	87.1%		91.0%	86.7%		88.0%	94.3%	
Don't know	2.2%	1.6%	5.1%		1.8%	4.7%		2.8%	1.0%	
Has your baby been bathed yet?										
No	92.6%	93.3%	89.3%	<0.00	93.3%	87.5%	<0.00	92.6%	92.6%	0.723
Yes	5.8%	5.9%	5.6%		5.9%	5.5%		5.6%	6.2%	
Don't know	1.6%	0.8%	5.1%		0.8%	7.0%		1.8%	1.2%	
After birth, did any health care provider examine the cord?										
No	28.0%	27.4%	30.9%	0.344	27.9%	28.9%	0.809	34.8%	18.0%	<0.001
Yes	72.0%	72.6%	69.1%		72.1%	71.1%		65.2%	82.0%	
After birth, did any health care provider assess the temperature of your baby?										
No	51.1%	52.6%	43.8%	0.033	52.0%	44.5%	0.113	56.3%	43.1%	<0.001
Yes	48.9%	47.4%	56.2%		48.0%	55.5%		43.7%	56.9%	
After birth, did any health care provider counsel you on danger signs for newborns?										
No	63.4%	63.4%	63.5%	0.983	63.4%	63.3%	0.974	73.0%	49.0%	<0.001
Yes	36.6%	36.6%	36.5%		36.6%	36.7%		27.0%	51.0%	
**Breastfeeding care indicators**										
Within two hours of delivery, did a health provider check if breastfeeding was going well.										
No	24.2%	22.8%	30.3%	0.048	23.9%	25.8%	0.248	29.8%	15.8%	<0.001
Yes	75.6%	77.0%	69.1%		76.0%	73.4%		69.9%	84.2%	
Don't know	0.2%	0.1%	0.6%		0.1%	0.8%		0.3%	0.0%	
While you were in the hospital for the delivery of this baby did any one counsel/talk to you about breastfeeding?										
No	34.6%	33.5%	39.9%	0.103	34.%	39.1%	0.258	40.7%	25.4%	<0.001
Yes	65.4%	66.5%	60.1%		66.%	60.9%		59.3%	74.6%	
While you were in the hospital for the delivery of this baby did any one help you with breastfeeding by observing or showing you how to breastfeed?										
No	42.4%	40.6%	50.9%	0.013	41.5%	48.8%	0.126	51.1%	29.6%	<0.001
Yes	57.6%	59.4%	49.1%		58.5%	51.2%		48.9%	70.4%	

Note: For analyses, clinical quality and breastfeeding care indicators, "don't know" responses were recoded as "no," and one missing response was conservatively recoded as "yes."

For peer review only - http://bmjopen.bmj.com/site/about/guidelines.xhtml

Results of selected multivariate logistic regression analyses (full models)

	Newborn separated for more than 10 minutes		Consent sought for newborn procedures					
	Exclusive breastfeeding at 2-4 week follow-up (n=829)		Satisfaction with newborn care at baseline (n=1,013)		Attend postpartum visit by either follow-up (n=1,013)		Exclusive breastfeeding at 10 week follow-up (n=839)	
Factor	Coeff	95%CI	Coeff	95%CI	Coeff	95%CI	Coeff	95%CI
**Baby separated > 10 min (ref. not separated/separated <=10min)**	0.560***	(0.404, 0.776)	---		---		---	
**Consent sought for newborn procedures (ref. consent not sought)**	---		2.713***	(1.668, 4.411)	1.274*	(1.050, 1.546)	1.334**	(1.100, 1.619)
Age (continuous)	1.106***	(1.050, 1.165)	0.996	(0.948, 1.046)	1.048*	(1.010, 1.089)	1.046*	(1.002, 1.092)
Married (ref. not married)	0.634	(0.336, 1.199)	0.837	(0.568, 1.231)	0.942	(0.664, 1.336)	0.785	(0.408, 1.511)
Education (ref. No education/primary)								
Vocational/Secondary	1.093	(0.797, 1.498)	1.176	(0.714, 1.937)	1.084	(0.869, 1.353)	1.963**	(1.194, 3.226)
College/University	0.809	(0.513, 1.277)	1.324	(0.679, 2.584)	1.427	(0.927, 2.196)	2.306	(0.961, 5.532)
Employed (ref. not employed)	0.658**	(0.452, 0.957)	0.502*	(0.267, 0.945)	0.792*	(0.641, 0.979)	0.751	(0.561, 1.007)
Born in Nairobi or Kiambu counties (ref. woman born elsewhere)	1.117	(0.764, 1.632)	0.506	(0.274, 0.934)	1.352*	(1.071, 1.707)	0.910	(0.597, 1.387)
Multiparous (ref. primiparous)	1.420	(0.971, 2.076)	1.378	(0.839, 2.262)	1.087	(0.768, 1.539)	1.445	(0.803, 2.602)
Health status (ref. Excellent/Very good)								
Good	0.979	(0.701, 1.367)	1.311	(0.762, 2.257)	0.886	(0.731, 1.074)	0.864	(0.537, 1.390)
Fair	1.092	(0.453, 2.634)	1.667	(0.769, 3.614)	0.907	(0.657, 1.252)	0.857	(0.480, 1.532)
Poor or very poor	0.738	(0.340, 1.603)	8.688	(1.977, 38.186)	0.752	(0.524, 1.079)	1.443	(0.953, 2.186)
Facility level (ref. Gov't Hospital)								
Gov't HC/Disp	1.195	(0.618, 2.309)	1.770***	(1.528, 2.052)	1.139	(0.691, 1.879)	0.791	(0.556, 1.124)
Private Facility	0.547	(0.281, 1.063)	1.655	(0.878, 3.120)	1.707*	(1.087, 2.679)	0.492***	(0.334, 0.725)
Covered by health scheme or health insurance (ref. not covered)	1.401	(0.899, 2.184)	1.700	(0.823, 3.514)	0.989	(0.741, 1.321)	1.093	(0.793, 1.508)
Maternal Delivery Complications (ref. no complications)	---		0.673	(0.311, 1.454)	---		---	
Newborn Complications at baseline (ref. no complications)	0.441**	(0.264, 0.738)	1.094	(0.714, 1.678)	---		0.891	(0.399, 1.988)
Maternal Complications since discharge (ref. no complications)	0.557***	(0.434, 0.715)	---		1.650*	(1.060, 2.568)	0.662	(0.410, 1.069)
Newborn Complications since discharge (ref. no complications)	0.782	(0.396, 1.544)	---		---		0.898	(0.454, 1.776)
Newborn Complications at either baseline or since discharge (ref. no complications)	---		---		0.998	(0.608, 1.638)	---	
Clinical care Index (continuous)	1.112	(0.958, 1.290)	1.843***	(1.581, 2.149)	1.307***	(1.198, 1.425)	1.015	(0.838, 1.230)
Breastfeeding care Index (continuous)	1.164	(0.853, 1.590)	---		---		1.092	(0.897, 1.330)
Constant	0.621	(0.193, 1.994)	0.958	(0.355, 2.588)	0.0690***	(0.027, 0.175)	0.930	(0.382, 2.264)

*p<0.05, **p<0.01, ***p<0.001

For peer review only

STROBE Statement—Checklist of items that should be included in reports of ***cohort studies***

	Item No	Recommendation	Page No
**Title and abstract**	1	(*a*) Indicate the study's design with a commonly used term in the title or the abstract	1,2
		(*b*) Provide in the abstract an informative and balanced summary of what was done and what was found	2
**Introduction**			
Background/rationale	2	Explain the scientific background and rationale for the investigation being reported	3-7
Objectives	3	State specific objectives, including any prespecified hypotheses	7
**Methods**			
Study design	4	Present key elements of study design early in the paper	8
Setting	5	Describe the setting, locations, and relevant dates, including periods of recruitment, exposure, follow-up, and data collection	8-9
Participants	6	(*a*) Give the eligibility criteria, and the sources and methods of selection of participants. Describe methods of follow-up<br>(*b*) For matched studies, give matching criteria and number of exposed and unexposed	8-9
Variables	7	Clearly define all outcomes, exposures, predictors, potential confounders, and effect modifiers. Give diagnostic criteria, if applicable	9-11
Data sources/ measurement	8*	For each variable of interest, give sources of data and details of methods of assessment (measurement). Describe comparability of assessment methods if there is more than one group	9-11
Bias	9	Describe any efforts to address potential sources of bias	10-11
Study size	10	Explain how the study size was arrived at	12, Supp 1
Quantitative variables	11	Explain how quantitative variables were handled in the analyses. If applicable, describe which groupings were chosen and why	9-10
Statistical methods	12	(*a*) Describe all statistical methods, including those used to control for confounding<br>(*b*) Describe any methods used to examine subgroups and interactions<br>(*c*) Explain how missing data were addressed<br>(*d*) If applicable, explain how loss to follow-up was addressed<br>(*e*) Describe any sensitivity analyses	10-11, Table footnotes, Supplement 1
**Results**			
Participants	13*	(a) Report numbers of individuals at each stage of study—eg numbers potentially eligible, examined for eligibility, confirmed eligible, included in the study, completing follow-up, and analysed<br>(b) Give reasons for non-participation at each stage<br>(c) Consider use of a flow diagram	9, Supp 1
Descriptive data	14*	(a) Give characteristics of study participants (eg demographic, clinical, social) and information on exposures and potential confounders<br>(b) Indicate number of participants with missing data for each variable of interest<br>(c) Summarise follow-up time (eg, average and total amount)	12-15, Table footnotes
Outcome data	15*	Report numbers of outcome events or summary measures over time	16-17

For peer review only

Main results	16	(*a*) Give unadjusted estimates and, if applicable, confounder-adjusted estimates and their precision (eg, 95% confidence interval). Make clear which confounders were adjusted for and why they were included<br>(*b*) Report category boundaries when continuous variables were categorized<br>(*c*) If relevant, consider translating estimates of relative risk into absolute risk for a meaningful time period	16-18
Other analyses	17	Report other analyses done—eg analyses of subgroups and interactions, and sensitivity analyses	11
**Discussion**			
Key results	18	Summarise key results with reference to study objectives	18-21
Limitations	19	Discuss limitations of the study, taking into account sources of potential bias or imprecision. Discuss both direction and magnitude of any potential bias	22-23
Interpretation	20	Give a cautious overall interpretation of results considering objectives, limitations, multiplicity of analyses, results from similar studies, and other relevant evidence	18-21
Generalisability	21	Discuss the generalisability (external validity) of the study results	23
**Other information**			
Funding	22	Give the source of funding and the role of the funders for the present study and, if applicable, for the original study on which the present article is based	24

*Give information separately for exposed and unexposed groups.

Note: An Explanation and Elaboration article discusses each checklist item and gives methodological background and published examples of transparent reporting. The STROBE checklist is best used in conjunction with this article (freely available on the Web sites of PLoS Medicine at http://www.plosmedicine.org/, Annals of Internal Medicine at http://www.annals.org/, and Epidemiology at http://www.epidem.com/). Information on the STROBE Initiative is available at http://www.strobe-statement.org.

For peer review only

For peer review only - http://bmjopen.bmj.com/site/about/guidelines.xhtml
